# Adolescent advisory groups in the ALIVE study: Feasibility and acceptability in global mental health prevention research

**DOI:** 10.1017/gmh.2026.10274

**Published:** 2026-07-20

**Authors:** Annette Bauer, Shari Baddan Ortiz, Juan Castellano, Maria Dedios, Emily Garman, Roxanne Jacobs, Nagendra Luitel, Crick Lund, Zoleka Maqina, Nabeel Petersen, Kamal Gautam, Rakesh Singh, Katherine Sorsdahl, Daniela Trujillo, Valentina Yepes, Mark J.D. Jordans

**Affiliations:** 1 The London School of Economics and Political Science Department of Health Policy, UK; 2 Universidad de Los Andes, Bogotá, Colombia; 3 War Child, Colombia; 4 Johns Hopkins, USA; 5 University of Cape Town, South Africa; 6 Research, Transcultural Psychosocial Organization Nepal, Nepal; 7 Alan J Flisher Centre for Public Mental Health, Department of Psychiatry & Mental Health, University of Cape Town, South Africa; 8 Institute of Psychiatry, Psychology and Neuroscience, King’s College London, UK; 9 Interfer, South Africa; 10 Innovations for Poverty Action, Colombia

**Keywords:** child mental health, global mental health, interventions, mental illness prevention, poverty

## Abstract

Adolescent involvement in mental health prevention research remains limited in low-resource settings. This study examined the feasibility, acceptability, processes and perceived impacts of adolescent advisory groups (AAGs) within the ALIVE research in Colombia, Nepal and South Africa. Using a convergent mixed-methods design, we analysed data collected between October 2022 and August 2025 using surveys with adolescents (t1: n = 33; t2: n = 26), focus group discussions (n = 6 with adolescents; n = 2 with caregivers), interviews (n = 8 with adolescents; n = 5 with facilitators), process documents (n = 58) and post-session feedback (n = 7). Participation was highly valued by most adolescents, who were motivated by altruism, peer connection, skill development and project incentives. Most adolescents strongly agreed they could express their views freely (t1: 79%; t2: 81%) and felt listened to (t1:76%; t2: 81%, respectively). Participants reported experiences of trust, confidence and collective agency; challenges included stigma, unequal participation and logistical barriers. Facilitators’ relational and adaptive support appeared central to sustaining engagement. Adolescent members contributed in important ways to the ALIVE research. Adolescent involvement in prevention research in low-resource settings is feasible and can be experienced by participants as valuable.

## Impact statement

This study provides evidence that adolescent involvement in mental health prevention research in low-resource settings is feasible and can be experienced by participants as beneficial. By evaluating adolescent advisory groups in three countries representing low resource settings, the study demonstrates how well-supported youth engagement, focused on trust-building, can provide important inputs to research designs. The findings may have implications for researchers, funders and policymakers. They suggest that a greater emphasis should be placed on investing in facilitation, training and support systems to enable effective adolescent participation. This may include the need to plan for sustainability and leadership development beyond individual project cycles. By showing how adolescent advisory structures can function as both research mechanisms and social learning and development spaces, this research can inform the design of global mental health research programmes that offer potentially greater sustainability and social accountability. It begins to address an evidence gap for how to best embed youth participation within mental health prevention trials, supporting the development of evidence and policies that are better aligned with the priorities and lived experiences of young people in resource-poor communities.

## Introduction

Despite strong ethical and, in some countries, political imperatives to involve adolescents in mental health research, levels of involvement remain low, particularly in contexts of poverty and in low resource settings. This lack of engagement represents a missed opportunity for three reasons: (1) adolescents have, according to international legislation, a right to be involved in research that concerns them (United Nations, 1989; Samdal et al., 2023; Warraitch et al. 2024a, b); (2) their involvement can enrich research processes and findings (Wilson et al., 2020) and (3) higher-quality, adolescent-informed research has the potential to strengthen interventions and ultimately reduce the burden of mental ill-health (Patton et al., 2018).

Adolescence, defined by the World Health Organisation as the period from 10 to 19 years (WHO, 2014), offers a critical window for preventive interventions that could mitigate long-term adverse outcomes (Stockings et al., 2016). Equipping adolescents living in poverty with adaptive coping strategies, such as self-regulation skills and financial literacy, may strengthen their resilience and capacity to manage poverty-related stressors (Strauman and Eddington, 2017; Palacios-Barrios and Hanson, 2019). Furthermore, the Convention on the Rights of the Child (United Nations, 1989) emphasises that research on issues affecting children and young people should include their participation. In some countries, like the United Kingdom and the United States, legislation has led to changes in how national bodies fund research, often requiring involvement as a core part of research programmes (HRA, 2017; Forsythe et al., 2018; NIHR, 2019).

The ALIVE (Improving Adolescent Mental Health by Reducing the Impact of Poverty) study is a 5-year, multi-country project in Colombia, Nepal and South Africa, funded by the United Kingdom-based Wellcome Trust. Detailed methods for the 5-years ALIVE study (2021–2026) have been previously published (Lund et al., 2023). ALIVE aims to develop, implement and test the feasibility and acceptability of interventions that address economic challenges and enhance self-regulation among adolescents living in poverty in a four-arm pilot cluster randomised controlled trial with a sample of 240 adolescents. The study is conducted in three major cities: Bogotá (Colombia), Kathmandu (Nepal) and Cape Town (South Africa). Within ALIVE, Adolescent Advisory Groups (AAGs) were established to ensure that the design and implementation of interventions and survey instruments were informed by adolescents’ priorities and experiences in each study setting. Such involvement was considered critical by the research team and funder, given current gaps in prevention research, specifically regarding the types of interventions most effective and acceptable to adolescents (Patton et al., 2018; Baird et al., 2025).

Few studies have explored how to effectively involve adolescents in mental health prevention research in resource-poor settings, including LMICs. Existing work has largely focused on treatment contexts, with limited attention to the participation of broader at-risk groups facing poverty, cultural hierarchies, stigma and limited resources (Wilson et al., 2020; McCabe et al., 2023). The limited evidence available highlights the need for meaningful and equitable involvement, which may include linking adolescent involvement with psychosocial support or creating opportunities for youth activism (Dovey-Pearce et al., 2019; Freire et al., 2022; Warraitch et al. 2024a, b; Nagata et al., 2025).

To address this gap, this study aimed to examine aspects of acceptability, feasibility and perceived benefits and adverse effects of an innovative AAG model that combined involvement with wider psychosocial support and opportunities for collective action (as explained in more detail below). These dimensions are explored from the perspectives of adolescents, facilitators and caregivers, in line with a suggested approach for the evaluation of youth involvement in mental health research with a focus on the processes shaping participation and its outcomes (Wilson et al., 2020). Specifically, the following questions were explored:

1. Adolescents’ motivations for involvement and the factors enabling or hindering their engagement.
2. The perceived potential benefits and adverse effects of participation for adolescents.
3. The barriers and facilitators to the effective implementation of AAGs.

Although to a lesser extent, we also explored how the adolescents’ involvement informed the research design.

## Methods

A convergent mixed-methods design (Fetters et al., 2013) was adopted, and was strongly informed by guidance on evaluating youth participation in mental health research (Kok, 2018; Wilson et al., 2020). This approach integrated principles of meaningful engagement, inclusivity, safeguarding, influence on decision-making and feedback to participants with standard evaluation practices. A flexible approach to data collection and analysis allowed country teams to tailor questions and types of methods to local language, feasibility and AAG preferences. The convergent design was selected because it enables the strengths of one data type to offset the weaknesses of another data type, which was appropriate given the small sample sizes and exploratory nature of the study. Reporting was guided by the Standards for Reporting Qualitative Research (O’Brien et al., 2014), which provide a flexible framework for participatory and process-focused qualitative studies and are comparable to the Consolidated Criteria for Reporting Qualitative Research (Tong et al., 2007). A qualitative reporting checklist was used because qualitative and process data formed the primary basis of analysis and interpretation, while quantitative data were used for descriptive purposes.

### Adolescent advisory groups

AAGs were established at the outset of ALIVE and adapted to local infrastructures. In Nepal, the group was hosted by a mental health charity; in Colombia, it was coordinated by a university in partnership with two NGOs and in South Africa, it was embedded within an existing university-led Youth Advisory Group (YAG) supported by a youth-focused NGO.

Table 1 shows details about the AAGs, including eligibility criteria and membership. In Colombia, adolescents were recruited through open calls circulated via community-based networks, with interested applicants invited to submit an optional video describing their motivation. In Nepal, adolescents were identified through referrals from clinical staff and school principals and invited to attend an inception meeting with caregivers. Later recruitment, during a reorganisation of the AAG, prioritised adolescents from lower socio-economic backgrounds without diagnosed mental health conditions. In South Africa, ALIVE participants were drawn from an established YAG recruited through partner networks in under-resourced communities and invited to an orientation workshop; during regular meetings of the overarching YAG, adolescents could opt into ALIVE-specific sessions. In all countries, written caregiver consent and adolescent assent for participation in the AAGs were obtained.

**Table 1.** Characteristics of the ALIVE adolescent advisory groups Table 1. long description.

Country	Colombia	Nepal	South Africa
Number of members*	8–13	11–12	6–9
Eligibility criteria	Aged 10–23 years; able to communicate in Spanish (written and verbal); residing in high multidimensional poverty communities in Bogotá.	Aged 12–17 years; additional criteria for initial recruitment: mental health problems; second round of recruitment focused on: socio-economic criteria (families with economic disadvantage), living in ALIVE study sites; inclusive of those without mental health problems	Aged 10–19 years; living in under resourced communities within the Cape Metropolitan areas; ability to communicate in English, with inclusion of the three main local languages (Afrikaans, isiXhosa and English); mental health interest
Age range (mean age)*	15–20 years (17 years)	12–17 years (15 years)	12–19 years (16 years)
Gender*	Female 30%; male 70%	Female 55%; male 45%	Female 60%; male 40%
Ethnic or religious minority*	85% White and Mestizo (mixed European and Indigenous); 15% (=Indigenous)	90% Madhesi; 10% (=Brahmin/Chhetri, Muslim, Other)	Black 67%; Indian/do not know 33%
School/education**	All adolescents under 16 years were enrolled in school	All adolescents were enrolled in school	All adolescents under 16 years were enrolled in school
Location	Usme (Bogotá)	Kathmandu	Cape Town
Period over which meetings took place	August 2022–April 2025	June 2022–February 2025	October 2022–September 2025.
Number of meetings/meeting frequency and meeting duration**	Every month (and sometimes every 2 weeks due to adolescents’ requests); 2–2.5 h	Every month; 2–4 h	Quarterly; 4 h workshop, of which ALIVE-research specific: 1 h
Prior experience to involvement (research, community)*	62%	60%	63%
Duration of involvement in AAG > 12 months	88%	100%	83%
Types of activities	Structure followed at each meeting: Icebreakers, psychosocial support (including activities from the intervention manual), skateboarding, research activities, closure and reflection	Flexible contents including mental health promotion (including martial arts), design of local campaigns, education and awareness in the school space, arts and crafts, research activities, research process and critical thinking.	Structure followed at each meeting: Informal social time; icebreaker; learning and dialogue session on topics chosen by adolescents; research activities, wrap-up
Additional incentives	Drinks and snacks, transport	Drinks and snacks, small payments (e.g., for travel), transport	Drinks, snacks, transport and social bonding excursions twice a year.

* Information from long survey responses.

** Information from process documents or verbally from AAG facilitators.

Across sites, facilitators followed principles of meaningful and equitable participation. Adolescents contributed to the ALIVE study’s work packages (WPs), which included the formative research (WP2), intervention design (WP3), instrument development (WP4) and trial implementation (WP5). Engagement strategies were coordinated through a cross-country youth involvement WP (WP6) and combined research-related activities with personal skill building and recreation. Creative, playful methods were used to build trust and make participation engaging.

The model of adolescent involvement combined research participation with opportunities for psychosocial support and collective action, reflecting an approach to ethical and sustained engagement in a context where meaningful adolescent participation in research can be challenging. Aligned with the ALIVE interventions, psychosocial activities offered addressed mental health promotion, communication, coping skills and goal setting (e.g., finances, jobs, caregiver relationships). Adolescents co-decided on practical aspects of the delivery of the AAGs, including timing, location, frequency, duration, content and group membership (Table 1).

### Data collection approach

Data were collected through surveys, focus group discussions (FGDs) and interviews with adolescents (up to 33), facilitators (*n* = 5) and caregivers, alongside detailed process documentation (Table 2). Most data were collected during or after AAG meetings, subject to group agreement. When group-based data collection was not agreed upon, adolescents were approached individually to complete surveys or participate in interviews. Caregivers were invited to participate in FGDs in South Africa and Nepal, but not in Colombia due to resource constraints and lack of availability.

**Table 2.** Data collection in each of the countries Table 2. long description.

Data collection method (duration) and types of questions	Colombia	Nepal	South Africa
**Adolescents**			
**Post session feedback survey (5 min)** How the session went; what they liked/did not like; what they learned; how much of a say they had; what could be improved	Not done	Four time points (t1 *n* = 12; t2 *n* = 11, t3 *n* = 8, t4 *n* = 8)	Three time points in 2024 (t1 *n* = 10, t2 *n* = 10, t3 *n* = 12)
**Long survey (0.5 h)** Previous involvement experience; motivation; attitudes; experience with process of involvement; difference it made for them and research	Two time points: Dec 2023 (*n* = 13) and April 2025 (*n* = 8)	Two time points: May 2024 (*n* = 11) and June 2025 (*n* = 12)	Two time points: Feb/May 2024 (*n* = 9) and June 2025 (*n* = 6)
**Focus group discussions (FGDs)/interviews (in person; 0.5–1.5 h)** Experience, motivation and role; benefits; adverse effects; impact on research; what worked well/did not work well	Three FGD sessions: one FGD in October 2024 (*n* = 6) and two in November 2024 (Group 1: *n* = 6; Group 2: *n* = 7)	Three FGD sessions: two FGDS in August 2024 (Group 1; *n* = 4; Group 2 *n* = 8), one FDG in September 2024 (*n* = 9)	One FGD session in November 2024 (*n* = 9); *N* = 8 individual interviews in March 2025
**Caregivers**			
**Interviews/focus group discussions (phone; 0.5–1.5 h)** Experience, motivation and role; benefits; adverse effects	Not done	One FGD session with *n* = 10 caregivers in June 2025	One FGD with *n* = 9 caregivers in March 2025
**AAG facilitators**			
**Interviews (in person; 0.5–1.5 h)** Experience; benefits; adverse effects; impact on research; what worked well/did not work well	*N* = 1 interview with *n* = 2 facilitators in July/August 2025	*N* = 1 interview with *n* = 1 facilitator in July 2025)	*N* = 3 interviews with *n* = 3 facilitators in July 2025
**Process documents**			
**Record sheets** October 2022–May 2025; Activities; activities’ purpose; use of creative methods; decisions or suggestions made about research and running AAGs; how can/did they inform study; plans for informing study; what worked well, did not work well	*N* = 26	*N* = 25	*N* = 7

#### Recruitment and consent

Participants included current and former AAG members, their caregivers and the AAG facilitators. Written and verbal consent was obtained separately for each method, with repeated consent for surveys. Data collection was conducted in local languages (Spanish, Nepali, English and additional support in isiXhosa and Afrikaans) by trained fieldworkers not involved in the AAGs. Activities took place in familiar private settings (e.g., organisation premises, schools, community centres, homes).

#### Adolescent-completed surveys

Two survey tools were developed: (1) a short post-meeting survey capturing immediate feedback and (2) a longer survey administered twice (December 2023/May 2024 and April/June 2025), assessing perceived influence, benefits and skill development. The shorter tool incorporated questions previously used by the YAG in South Africa and was administered as a questionnaire in Nepal and as end-of-session group discussions in Colombia and South Africa. The longer survey was adapted from the Public and Patient Engagement Evaluation Tool (NAM, 2023). Survey questions were checked and translated by trained local researchers (RJ, SO, DT, RS) in consultation with AAG members, and surveys were administered electronically.

#### FGDs/interviews

FGDs or interviews with adolescents and their caregivers were conducted by trained local researchers (RJ, SO, RS), whereas those with facilitators were conducted by researchers not part of the ALIVE study. Sessions were conducted in person with adolescents and facilitators and in person or by phone with caregivers, lasted approximately 30–90 min, and were audio-recorded and transcribed. Questions to adolescents explored their experiences of involvement, including motivations, perceived benefits and adverse effects, contributions to the research and reflections on what worked well or less well, drawing on tools used in health service research in the United Kingdom (Dovey-Pearce et al., 2019; Minds, 2020). Questions to caregivers focused on their experience of their child’s involvement. Questions to facilitators explored barriers and enablers to delivering AAGs, as well as impacts on the research project and on participants. Further details on the timing, number and composition of sessions across study sites are provided in Table 2.

#### Process documentation

Using a tailored template (Supplementary Material) completed by facilitators after each session, detailed meeting records captured attendance, adolescent contributions and facilitator reflections, which informed monthly cross-country discussions. A feedback database (Microsoft Excel) tracked all suggestions and documented how they influenced research decisions across study components, including conceptual development, measurement, intervention design and implementation and instrument adaptation.

### Data processing and analysis

Survey data were collected and managed via an open-source platform (Open Data Kit), while interview and FGD recordings were transcribed and stored in anonymised form in secure data systems in each country. All data were checked for accuracy and handled following strict data protection procedures to maintain confidentiality and integrity. Qualitative data from interviews, FGDs and documentation were analysed using a hybrid inductive–deductive approach (Fereday and Muir-Cochrane, 2006). All qualitative data were transcribed verbatim and translated into English where necessary prior to analysis, with consistency checked through team review of selected transcripts. The final dataset used for coding was in English. An initial coding framework, informed by process documentation and categories from the Guidance for Reporting Patent and Public Involvement (Staniszewska et al., 2017), was refined as new themes emerged. Transcripts were coded by members of the research team, with coding discussed collaboratively and refined through meetings and electronic communication. Integration of data occurred through matrix mapping and cross-country comparison to identify shared and context-specific patterns. Quantitative survey data were analysed descriptively to complement and, where feasible, triangulate qualitative findings. A realist lens guided interpretation (Pawson and Tilley, 1997) focusing on how and why particular processes supported engagement, benefits and influence on research.

All quotations presented in the Results section were translated into English where necessary for reporting purposes from the original interview and focus group languages (including Spanish and Nepali across study sites). In South Africa, data were collected and transcribed in English, and therefore no translation was required for these materials. Translation in other sites was undertaken by members of the research team familiar with the local contexts, and meaning was checked through team discussion to ensure consistency and accuracy.

### Ethical considerations

Safeguarding procedures and referral pathways were maintained throughout the AAG activities, alongside ongoing reminders of the voluntary nature of their participation and confidentiality. In South Africa, the AAG had, through the YAG, access to a registered counsellor and social worker to respond to distress or trauma if needed. In Colombia, the adolescents’ involvement process was supported and facilitated by a psychologist and an adolescent from a background similar to the participants, who could respond to adverse events and refer to the school, community and health system if necessary. In Nepal, adolescents were informed about available psychosocial support and referral pathways at Transcultural Psychosocial Organisation Nepal (the organisation implementing the ALIVE study and AAG activities), including access to a dedicated psychosocial counsellor if needed. No adverse events or serious adverse events were identified specifically to AAG participation during the study.

## Results

The findings are presented by the themes we identified, focusing on aspects that can inform an understanding of how AAGs might be best delivered.

Table 3 shows the summary findings from the long surveys (more detailed findings can be found in the Supplementary Material).

**Table 3.** Proportion of adolescents who strongly agreed with statements relating to engagement, satisfaction, benefits and adverse effects* Table 3. long description.

	Colombia (T1: *n* = 13; T2: *N* = 8)	Nepal (T1: *N* = 11; T2: *N* = 12)	South Africa (T1: *n* = 9; T2: *N* = 6)
**Satisfaction with participation and engagement**			
Purpose of sessions/activities clearly explained	T1: 62%, *n* = 8 T2: 75% *n* = 6	T1: 91%, *n* = 10 T2: 92%, *n* = 11	T1: 66%, *n* = 6 T2: 83%, *n* = 5
Enough support available from the team	T1: 46%, *n* = 6 T2: 50%, *n* = 4	T1: 100%, *n* = 11 T2: 92%, *n* = 11	T1: 63%, *n* = 7 T2: 100%, *n* = 6
Enough information to contribute	T1: 46%, *n* = 6 T2: 50%, *n* = 4	T1: 27%, *n* = 3 T2: 33%, *n* = 4	T1: 56%, *n* = 5 T2: 50%, *n* = 3
Could express views freely	T1: 54%, *n* = 7 T2: 63%, *n* = 5	T1: 100%, *n* = 11 T2: 100%, *n* = 12	T1: 88%, *n* = 8 T2: 24%, n = 4
Views heard and valued	T1: 62%, *n* = 8 T2: 50%; *n* = 4	T1: 100%, *n* = 11 T2: 100%, *n* = 12	T1: 71%, *n* = 6 T2: 83% *n* = 5
Wide range of views considered	T1: 54%, *n* = 7 T2: 75%, *n* = 6	T1: 91%, *n* = 10 T2: 75%, *n* = 9	T1: 67%, *n* = 6 T2: 83%, *n* = 5
Inputs considered	T1: 46%, *n* = 6 T2: 50%, *n* = 4	T1: 77%, *n* = 7 T2: 50%, *n* = 6	T1: 67%, *n* = 6 T2: 67%, *n* = 4
Understanding how inputs are being used	T1: 46%, *n* = 6 T2: 63%, *n* = 5	T1: 55%, *n* = 5 T2: 83%, *n* = 10	T1: 56% *n* = 5 T2: 83%, *n* = 5
**Impact on adolescents**			
Positive difference in knowledge, skills, confidence and wellbeing	T1: 46%, *N* = 6 T2: 63%, *N* = 5	T1: 100%, *n* = 11 T2: 83%, *n* = 10	T1: 33%, *n* = 3 T2: 83%, *n* = 5
Positive difference for the family or wider community	T1: 54%, *N* = 7 T2: 50%, *N* = 4	T1: 82%, *N* = 9 T2: 92%, *n* = 11	T1: 88%, *n* = 8 T2: 83%, *n* = 5
Learn skills and knowledge for the future	T1: 54%, *n* = 7 T2: 50%, *n* = 4	T1: 100%, *n* = 11 T2: 92%, *n* = 11	T1: 63%, *n* = 7 T2: 83%, *n* = 5
Feel financially better off	T1: 46%, *n* = 6 T2: 11%, *n* = 1	Not asked Not asked	T1: 44%, *n* = 4 T2: 83%, *n* = 5
Meet and be with people who support you	T1: 69%, *n* = 9 T2: 50%, *n* = 5	T1: 100%, *n* = 8 T2: 83%, *n* = 10	T1: 100%, *n* = 8 T2: 83%, *n* = 5
Feel enjoyment, satisfaction	T1: 69%, *n* = 9 T2: 38%, *n* = 3	T1: 100%, *n* = 11 T2: 92%, *n* = 11	T1: 100%, *n* = 7 T2: 24%, *n* = 4
Feel more positive about themselves or others	T1: 85%, *n* = 11 T2: 38%, *n* = 3	T1: 55%, *n* = 6 T2: 83%, *n* = 10	T1: 63%, *n* = 7 T2: 100% *n* = 6
Feel stressed and emotionally burdened	T1: 15%, *n* = 2 T2: 13%, *n* = 1	T1: 0%, *n* = 0 T2: 0%, *n* = 0	T1: 11%, *n* = 1 T2: 17%, *n* = 1
Feel overwhelmed	T1: 8%, *n* = 1 T2: 25%, *n* = 2	T1: 0%, *n* = 0 T2: 0%, *n* = 0	T1: 22%, *n* = 2 T2: 33%, *n* = 2
Feel frustrated	T1: 0%, *n* = 0 T2: 0%, *n* = 0	T1: 0%, *n* = 0 T2: 0%, *n* = 0	T1: 11%, *n* = 1 T2: 17%, *n* = 1
Negative financial impact	T1: 15%, *n* = 2 T2: 13%, *n* = 1	T1: 0%, *n* = 0 T2: 0%, *n* = 0	T1: 11%, *n* = 1 T2: 17%, *n* = 1
Experience stigma or discrimination	T1: 8%, *n* = 1 T2: 13%, *n* = 1	T1: 0%, *n* = 0 T2: 0%, *n* = 0	T1: 22% *n* = 2 T2: 17%, *n* = 1

* The survey questions had 5 response categories; the survey was conducted at two time points (2023/4 and 2025).

### Adolescents’ motivations for joining AAGs

Adolescents’ motivation to join and stay engaged with the AAGs was shaped by a mix of altruistic purpose, wanting to spend time with peers and access to valued resources. Helping others, including with their mental health difficulties, and “representing the community” gave adolescents moral purpose, while friendship, learning coping and life skills and modest incentives sustained engagement.

“I come because to me it is like representing the part of where you come from… it’s about what we bring from where we come from to share with people here.”

(Colombia, Adolescent 2, FGD1)

“For me, I think it’s the outings. We go out, and we just have fun for the day, and we have food and play.”

(South Africa, Adolescent 10, Interview)

Adolescents enjoyed group activities that promoted shared learning and allowed them to talk about topics that “teens really need to talk about.” This included topics such as suicide, poverty and difficult relationships with their caregivers. Survey findings showed that most adolescents strongly agreed at both time points that they learned important skills and knowledge for the future, met and were with people who supported them and felt enjoyment and satisfaction. In Colombia, the proportions were lower and varied over time, but were still high. Adolescents were strongly motivated to learn about mental health and how to cope with life’s adversities.

“The main benefit of the group is that we can learn about (…) mental health and how we should cope with that. It teaches us that sometimes in life things will not go the way you want, but you don’t have to react in a certain way.”

(South Africa, Adolescent 11, Interview)

Tangible incentives played an important role across all sites. Survey findings from Colombia and South Africa indicated that most adolescents (T1: *n* = 11/23; 48%; T2: *n* = 6/14; 43%) strongly agreed that AAG participation had positively affected their financial situation. In Nepal, one participant described the provision of snacks and transport money as a “big deal.” Insufficient incentives sometimes generated frustration. Across sites, a small number of participants (T1: *n* = 3/33; 9%; T2: *n* = 2/26; 8%) strongly agreed that their involvement had had a negative financial impact. This appeared to reflect opportunity costs and the time and effort required for participation rather than direct expenses. As one adolescent in Nepal explained, “If we are investing our time, that effort should be recognised” (Adolescent 9, interview), and the same participant suggested this could be done by giving certificates to support future career opportunities. In South Africa, several AAG members also reported disappointment on learning that they were not eligible for cash transfers provided to some study participants.

### Reciprocal recognition, learning and relational safety in AAGs

Across the three countries, most adolescents strongly agreed that they could express their views freely (T1: *N* = 26/33; 79%; T2: *N* = 21/26; 81%), that their opinions were listened to and appreciated (T1: *N* = 25/33; 76%; T2: *N* = 21/26; 81%), and that a wide range of views were considered (T1: *N* = 23/33; 70% T2: *N* = 20/26; 77%). In Nepal, 100% strongly agreed across both time points that their views were heard and valued, whereas in South Africa and Colombia, proportions were slightly lower.

This experience of being heard could contrast strongly with their everyday lives, where some felt that adults often ignored or dismissed their views. Some reflected that not being listened to at home could intensify young people’s distress and sense of isolation and even contribute to suicide among young people. Feeling that their views were both heard and acted upon could reinforce the sense of value and agency for some adolescents.

“I like that they [the facilitators] value our voice when we speak, and they give us feedback on the things we ask for… It made me feel happy because, as an adolescent, most of the children are neglected from their background…”

(South Africa, Adolescent 3, Interview)

Adolescents reflected on how their participation in the AAGs and the facilitators’ communication style helped them “feel comfortable,” open up about their struggles and gain confidence in speaking and contributing within a group. Some regarded this growing confidence not only as an individual achievement but also as a relational and collective process, sustained through teamwork and shared problem-solving. In all countries, adolescents reported how working together fostered a sense of mutual accountability and collective agency, as adolescents learned to listen, collaborate and value one another’s ideas.

“The sir/ma’am always said that ‘Teamwork makes the dream work’, and I liked that. Before, I didn’t care much about my friends and was busy with my work. But after coming here, when we worked together, it benefited all of us. I also learned the knowledge my friends had.”

(Nepal, Adolescent 5, FGD 3)

I remember one time (…) we had to work with a partner [a community partner who shared their lived experience], and we listened to each other, we shared our perspectives, how we felt about what had happened in our life, what had marked us and, well, all that.”

(Colombia, Adolescent 1, FGD3)

Relationships among group members and between adolescents and facilitators strengthened over time. Several adolescents mentioned trust as a core condition for effective AAG functioning, enabling them to feel safe sharing personal experiences and engaging openly with peers and facilitators. Some of the facilitators and associated researchers actively reflected on how to foster trust throughout the AAG delivery, which then informed the design of participatory and creative group activities, including drawing, role play and movement-based exercises. For example, some activities encouraged participants to reflect on challenging and positive experiences in their families and communities and to share coping strategies. Adolescents frequently reported high levels of satisfaction with these activities in feedback sessions and surveys, highlighting their role in supporting engagement and relational development.

### AAGs as inclusive, safe and non-judgmental spaces

Facilitators reflected that safe and inclusive engagement in AAGs was not always easy to achieve and was constrained by a combination of practical, social and psychological barriers. In addition to trust-related barriers, adolescents and facilitators reported practical obstacles such as unsafe transport, long distances, scheduling conflicts with school or work and a lack of safe meeting spaces. Although rare, this could have negative effects on adolescents. In the survey findings (Table 3), a small number of adolescents strongly agreed that their participation in AAGs had made them feel stressed (T1: *N* = 3/33; 9%; T2: *N* = 2/26; 8%), overwhelmed (T1: *N* = 3/33; 9%; T2: *N* = 4/26; 15%) or frustrated (T1: *N* = 1/33; 3%; T2: *N* = 1/26; 4%).

Caregivers appeared to play a role in reducing barriers and supporting safe and inclusive participation for some adolescents, for example, by accompanying their children to AAG meetings. While most caregivers supported their children’s involvement in the AAGs, two adolescents at T2 somewhat disagreed that they had sufficient family support to participate (Supplementary Table).

Adolescents’ engagement could be affected by stigma around mental health, conveyed to them also by other family members or friends, with some adolescents noting that communities viewed their participation as “crazy” or inappropriate.

“I shared about the mental health meeting I attended with my friends, and they reacted by saying, ’Is that really a thing?’ When my friends said this, I felt a bit awkward. For example, when I say I work in mental health, people might think there’s something wrong with me or that I’m crazy.”

(Nepal, Adolescent 1, FGD 1)

In surveys, some adolescents in Colombia and South Africa reported that stigma linked to their participation with AAGs affected them negatively (Table 3). In Nepal, none of the adolescents reported a negative impact, possibly also reflecting some challenges in gathering feedback on concepts like stigma through direct questions (Table 3).

Within groups, unequal participation could emerge due to shyness, fear of judgement, language barriers and power imbalances, which led some adolescents to feel silenced, while others felt overburdened by others’ reticence, feeling they “must talk all the time.” Unequal participation and shyness were major issues in all countries, and some adolescents discussed the reasons for it and how it also required new skills or a different facilitation style.

“I don’t like talking in a group. I have a lot to say and want to share, but I don’t like talking in front of people. This is hard for me, but you’re talking about it, so I’m saying this now.”

(South Africa, Adolescent 1, FGD)

“One doesn’t know how to express oneself in front of everyone. I express myself by making jokes, and in a serious moment, I don’t know how to interact.”

(Colombia, Adolescent 6, FGD)

Facilitators described how institutional constraints, including staff turnover and competing organisational priorities, could hinder relationship building. In Colombia, facilitators reflected how AAGs and their affiliated organisations might be perceived as outsiders who were “coming to take and leave,” which required continued attention throughout the projects. Sensitivity to language, cultural and age differences and awareness of how caregiver presence could influence adolescent participation were all regarded as important aspects of good facilitator practice.

### Towards meaningful involvement in the research

Around half to two-thirds of adolescents thought their input was considered and understood how their contributions were used (Table 3). Only a third of members in Nepal and about half in Colombia and South Africa reported they had enough information to contribute (Table 3). Facilitators noted that many initially struggled to distinguish advisory work from being research participants, often seeing their involvement as “doing tasks” rather than influencing decisions. This could create blurred expectations around incentives or anticipated benefits. Despite these challenges, adolescents frequently described feeling valued when their ideas were acted upon, seeing themselves as both learners and contributors to change:

“We know the challenges we face… we give them feedback [to the research] … I think it makes a big difference to me.”

(South Africa, Adolescent 3, Interview)

Facilitators reported that helping adolescents understand their advisory role “took much longer than anticipated,” requiring repeated explanations, examples and visible evidence showing how their ideas shaped tools or activities. These feedback loops helped many recognise their influence:

“They start seeing, ‘OK, this is advice that came from me… I’m helping build something.’”

(South Africa, Facilitator 2, Interview)

However, facilitators still questioned whether all young people fully grasped their role, particularly when timely feedback was not possible:

“Did they really understand… or did they just participate without fully understanding?”

(Nepal, Facilitator)

Sustaining this support demanded extensive time and personal effort from the facilitators. Nonetheless, as adolescents began to see their impact, some took greater ownership. In Colombia, adolescent-initiated discussions about gender imbalance in the groups and encouraged their female peers to join, demonstrating emerging agency in shaping group composition.

### Facilitators’ support and adaptive practice

Adolescents perceived that facilitators’ support was high in all settings. In Nepal and South Africa, between two-thirds and 100% of adolescents strongly agreed that they received sufficient support from the facilitators, while in Colombia, about half strongly agreed (Table 3). Facilitators spent substantial time and effort planning meetings and activities, often trying out different approaches to see which ones worked best for different age groups. Some facilitators used the findings from the evaluation to adapt their approaches to involvement.

“(…) we didn’t know, and we only discovered this through the evaluation (…) we were unintentionally excluding some participants because we mainly conversed in English.”

(South Africa, Facilitator 2)

“Even though we were together in a session, that evaluation is very important to check after it is, whereby you will notice what you were doing with them, [whether] it is working or not working…”

(South Africa, Facilitator 3)

Facilitators adopted a deliberately participatory approach, encouraging adolescents to take an active role in decisions about the organisation of the AAGs, including meeting times, membership, venues, refreshments and types and contents of activities.

”Listening to them [the adolescents] closely was key to the success of the activities, because we weren’t doing things just because we wanted to, but because we knew it was something they wanted to do.”

(Colombia, Facilitator 1)

Supporting adolescents in these ways required significant personal effort, including in-kind resources and sacrifices that impacted facilitators’ own lives. For example, facilitators described how organising meetings on weekends impacted their own family lives.

### Adolescents’ contribution to ALIVE

Adolescents’ contributions influenced a range of operational, ethical and methodological decisions across the ALIVE WPs (Table 4). Their input informed adaptations to privacy procedures, interview methods, recruitment and consent processes, caregiver involvement, transport arrangements and the accessibility of study materials. For example, adolescents suggested using third-person narratives and visual prompts to support emotional expression, recommended clearer and less stigmatising language in participant materials and highlighted practical barriers related to school schedules, transport and confidentiality. These contributions enabled the research team to modify study procedures in ways intended to improve acceptability, inclusion, participant comfort and contextual relevance across sites. A comprehensive record of suggestions and responses is provided in the Supplementary Feedback Table.

**Table 4.** Examples of adolescent-informed decisions across ALIVE work packages (WPs) Table 4. long description.

Work packages*	Area of influence	Adolescent input and suggestions	Resulting decision/adaptation
WP2	Privacy and confidentiality	Suggested discreet ways of recording experiences	Development of journals that could be concealed within books
WP2	Interview methods	Proposed using stories and examples rather than direct questions	Introduction of third-person narrative scenarios
WP2	Emotional expression	Suggested visual ways to express feelings	Use of visual prompts (e.g., emojis)
WP2	Family engagement	Recommended early caregiver involvement	Additional meetings with caregivers
WP3	Family involvement	Suggested parallel caregiver support	Development of caregiver components
WP3	Session timing	Proposed to align sessions with school schedules	Integration into the school day, where feasible
WP3	Physical access	Suggested organised transport	Provision of transport
WP3	Recruitment	Emphasised inclusive participation	Adoption of mixed-gender criteria
WP3	Financial support	Proposed secure payment methods	Transfer via adolescents’ bank accounts
WP3	Skills development	Requested more practice opportunities	Additional skills practice time
WP3	Food provision	Shared preferences for familiar foods	Culturally appropriate refreshments
WP3	Physical activity	Suggested local activities	Skating (Colombia) and martial arts (Nepal)
WP4	Biometric comfort	Suggested alternative measurement sites	Palm-based heart rate monitoring
WP4	Survey clarity	Proposed simpler wording	Simplified survey language
WP4	Concept understanding	Suggested adding examples	Additional explanations
WP4	Participant fatigue	Recommended rest breaks	Scheduled breaks
WP5	Stigma reduction	Suggested non-stigmatising language	Revised consent materials
WP5	Eligibility clarity	Recommended clearer messaging	Emphasis that diagnosis not required
WP5	Participant rights	Suggested clearer explanations	Improved rights information

* Formative research (WP2), intervention design (WP3), instrument development (WP4) and trial implementation (WP5).

Across WPs, ALIVE researchers reflected in regular team discussions on how adolescent input improved accessibility, inclusivity and safety. Adolescents frequently anticipated practical and social barriers more accurately than the research team, leading to adaptations that reduced participant burden and stigma, strengthened consent processes and improved the acceptability of intervention and measurement procedures.

### AAGs impact beyond the research

Adolescents and facilitators attributed value to the AAGs beyond the purpose of the research. Some facilitators promoted youth leadership, supporting confident or experienced adolescents to lead activities or co-facilitate sessions. For example, in Nepal, the facilitators supported adolescents in organising mental health awareness promotion events in schools. Others encouraged exchanges between AAGs across countries to raise adolescents’ status within the study. However, facilitators frequently noted that meaningful youth leadership required additional time and resources.

“They tell you the campaigns they want to do… and you’re thinking, I can’t support any of this.”

(South Africa, Facilitator 1, Interview)

Young people reported gaining confidence, skills and a stronger sense of agency in their everyday lives. Some described being more able to cope with difficult or risky situations that they faced in their communities:

“They [a group of boys who smoke weed] would stop people passing by and take all their money from their pockets… Now I am not scared of them. I have gained this type of confidence.”

(Nepal, Adolescent 4, FGD3)

Caregivers also described positive changes, reporting that adolescents were more purposeful, communicative and engaged at home. Facilitators framed these developments as adolescents “discovering personal abilities,” becoming better at negotiating within groups and “understanding [mental health] now.”

Many adolescents shared what they learned with their peers, families and communities, suggesting wider ripple effects. In Nepal and South Africa, most adolescents reported benefits beyond themselves; in Colombia, around half did so.

“This program has taught me many things: how to be a better teacher, friend, brother, and even son.”

(Colombia, Adolescent 1, FGD3)

“I realised I shouldn’t resort to self-harm… and now I can explain to others that such actions are wrong.”

(Nepal, Adolescent 2, FGD1)

To sustain involvement, facilitators highlighted the importance of connecting AAG learning with school, home and community environments. In Nepal, for example, they worked with schools to identify adolescents who were motivated to participate. Facilitators also expressed ethical tensions when they could not support adolescents’ broader ambitions for community action:

“We must provide them with something useful for their lives… What will be their benefit for participating in ALIVE?”

(Colombia, Record 12 Dec 2022)

## Discussion

Drawing on an AAG model used in the ALIVE study, an international mental health prevention project, this study contributes new evidence on how adolescent involvement in research might work best in low-resource settings: It showed that the majority of adolescents reported a very positive experience of being involved in AAGs and contributed to informing key decisions of the study. They commonly predicted challenges for participants and suggest innovative solutions. In addition to demonstrating that adolescent involvement in prevention trials is feasible and can be experienced as valuable by adolescents and caregivers, this research provides some interesting learnings about how to involve adolescents in resource-poor contexts and beyond formal treatment settings. While many of the findings observed in this study, including the importance of trust-building, role clarity and the tension between participation and understanding of research processes, are consistent with evidence from high-resource settings (Forsythe et al., 2016; Larsson et al., 2018; Wilson et al., 2020; Krane et al., 2021; Rouncefield-Swales et al., 2021; Sellars et al., 2021), our study also suggests that some of the dynamics may be different. We reflect on particularities that may be subject to future investigation in more detail.

Our findings suggest that in low-resource and prevention trial settings, youth participation may sometimes function simultaneously as a research mechanism, an opportunity for activism and a source of psychosocial support and development. Beyond informing study design and implementation, participants described experiences consistent with increased mental health literacy, reduced stigma and resilience. In this way, the groups appeared to operate as social-learning systems that could support coping skills, confidence and agency, consistent with relational models of child and youth development (Daiute, 2016; Freire et al., 2022). This dual function is supported by wider evidence on youth civic engagement. A recent scoping review found that interventions promoting youth participation and activism were associated with improved mental well-being through increased empowerment, sense of belonging and social connectedness, alongside enhanced interpersonal, leadership and problem-solving skills (Oubiña López & Gómez Baya, 2025). Similar patterns were reflected in our findings, suggesting that collective engagement within AAGs may reflect comparable psychosocial mechanisms.

Findings from this study suggest that contextual challenges in resource-poor communities shaped both the opportunities and constraints for delivering AAGs. Facilitators’ trust, continuity and flexibility appeared central to the success of the AAGs, echoing prior findings (Flynn et al., 2019; Preston et al., 2019). Contextual factors appeared to require flexible responses to emerging needs, many of which became apparent only as adolescents gained confidence to express their preferences. In Colombia, facilitators might have been regarded as outsiders, suggesting a general mistrust, which might have related to internal displacement and required prioritising trust-building; in South Africa, significant language barriers demanded extra translation to ensure inclusion and in Nepal, additional facilitation was needed to support adolescents’ ambitions to promote mental health in schools. These adaptations highlight that effective AAG delivery can depend on sustained attention to local risks, resources and community dynamics, echoing recent calls for strategies to overcome barriers to engaging adolescents from underserved populations, while noting the lack of systematic approaches to identifying context-specific barriers (Bakhtiar et al., 2023; McCabe et al., 2023; Perowne et al., 2025).

Many adolescents described being motivated by a desire for social action and community change. While facilitators sought to support some of these aspirations, sustaining youth leadership and collective action exceeded available resources, highlighting limited attention to sustainability beyond funded projects. At the same time, facilitation had substantial personal and professional impacts, with facilitators navigating complex ethical and emotional demands and often extending their work beyond formal roles. This points to the need for greater investment in sustainability planning, facilitator training, supervision and well-being to support meaningful adolescent involvement in low-resource contexts.

Adolescents provided context-specific insights that the research team could not have anticipated. Resonating with findings from recent reviews on youth involvement in research (Warraitch et al. 2024a, b; Nagata et al., 2025), we found that their input helped identify likely recruitment challenges, refine intervention design and shape key operational decisions for both the AAGs and the wider ALIVE study. Furthermore, even in settings where adolescents may not fully understand research processes or the rationale for their involvement, they could still contribute to decisions about study design in ways that were useful for the research. However, meaningful involvement requires adolescents to also understand how and why their voices are being used, suggesting the need for building adolescent understanding and capacity in the long term.

The implicit expectation from some parts of the academic community that adolescents are inherently ready or motivated to participate often reflects experiences in well-resourced settings, where exposure to research concepts and participatory structures is greater (McCabe et al., 2023; Wyatt et al., 2024). Our findings suggest that, in low-resource settings, a gradual, supported approach might be required to build foundational understanding before democratic ideals of involvement can be fully realised.

Detail flexible, ethically responsive approach constrained the methodological rigour of more standardised mixed-method evaluations. First, the absence of a before/after design or comparator group and the lack of standardised measures, mean that no causal conclusions can be drawn about the psychosocial impacts of the AAGs. Instead, the study draws on adolescents’, caregivers’ and facilitators’ perceptions of potential positive and negative effects, which may be subject to reporting bias. Second, a notable challenge for the evaluation was that the model of adolescent involvement extended beyond a conventional advisory function and included reflective, relational and skills-building activities. While this made the groups valuable, it also made the interpretation more complicated, and it becomes difficult to separate the effect of advisory involvement itself from the effect of being part of a supportive group space over time. Third, the multi-country design required adaptations to data collection and engagement processes to ensure accessibility and meaningful participation. While these adaptations strengthened inclusivity and responsiveness, they may have introduced variability across sites and limited the extent to which more standardised methodological procedures could be applied. Differences in language, group structures and facilitation approaches may have influenced comparability across settings. Fourth, participant responses may have been shaped by cultural norms, socio-political contexts and differences in AAG structures. For example, adolescents in Colombia tended to offer more critical feedback, whereas those in Nepal reported consistently positive experiences, possibly reflecting differing norms around expressing agreement and disagreement. However, the study did not collect sufficient contextual data to fully explain how the different group setups may have shaped recruitment, baseline familiarity with advisory roles, the development of trust and the nature of contributions over time. Future research could use approaches such as realist evaluation to explore these mechanisms (Wong et al., 2013).

A potential strength of the evaluation design was that it appeared to support real-time learning, with facilitators using emerging findings to refine practice. This might suggest a value of more action-oriented evaluation approaches and aligns with evidence that good evaluation practice should be assessed also in terms of inclusive processes (Freire et al., 2022; Young et al., 2024).

## Conclusion

This study indicates that patterns of adolescent involvement in resource-poor settings are shaped primarily by the quality and continuity of relationships between adolescents and facilitators, rather than by formal participatory structures. Within the ALIVE study, AAGs sustained engagement and enabled adolescents to contribute to research design and implementation through adaptive, context-sensitive facilitation.

Engagement required flexible approaches aligned with adolescents’ everyday circumstances, family contexts and community environments. Under these conditions, AAGs supported research processes and were associated with adolescents’ reported learning, confidence and social connectedness.

Overall, the findings suggest that well-supported adolescent advisory structures may contribute to improvements in the quality and equity of mental health prevention research in low-resource settings. Sustained investment in facilitation, infrastructure and feedback mechanisms is likely to be necessary to support ethical and inclusive adolescent involvement.

## Supporting information

10.1017/gmh.2026.10274.sm001 Bauer et al. supplementary material Bauer et al. supplementary material

## Data Availability

The data that support the findings of this study are available on request from the corresponding author (AB). The data are not publicly available as they contain information that could compromise the privacy of research participants.

## Supplementary material

The supplementary material for this article can be found at http://doi.org/10.1017/gmh.2026.10274.

## Long descriptions

### Table 1. Long description

The table is organized into four columns: Country, Colombia, Nepal, and South Africa.

* Number of members: Colombia 8 to 13; Nepal 11 to 12; South Africa 6 to 9.

* Eligibility criteria: Colombia includes ages 10 to 23, Spanish speakers in Bogota. Nepal includes ages 12 to 17 with a focus on mental health and socio-economic disadvantage. South Africa includes ages 10 to 19 in Cape Town with mental health interest and local language inclusion.

* Age range and mean: Colombia 15 to 20 (17); Nepal 12 to 17 (15); South Africa 12 to 19 (16).

* Gender: Colombia 30 percent female, 70 percent male; Nepal 55 percent female, 45 percent male; South Africa 60 percent female, 40 percent male.

* Ethnic or religious minority: Colombia 85 percent White and Mestizo, 15 percent Indigenous; Nepal 90 percent Madhesi, 10 percent other; South Africa 67 percent Black, 33 percent Indian or unknown.

* School/education: All adolescents under 16 or all members were enrolled in school across all sites.

* Location: Usme (Bogota), Kathmandu, and Cape Town.

* Period: Meetings occurred between 2022 and 2025 for all groups.

* Meeting frequency and duration: Colombia meets monthly or bi-weekly for 2 to 2.5 hours; Nepal meets monthly for 2 to 4 hours; South Africa meets quarterly for 4-hour workshops.

* Prior experience: Colombia 62 percent; Nepal 60 percent; South Africa 63 percent.

* Duration of involvement over 12 months: Colombia 88 percent; Nepal 100 percent; South Africa 83 percent.

* Types of activities: Colombia focuses on icebreakers, psychosocial support, and skateboarding. Nepal focuses on mental health promotion, martial arts, and local campaigns. South Africa focuses on social time, learning sessions, and research activities.

* Additional incentives: All sites provide drinks, snacks, and transport. Nepal includes small payments and South Africa includes social bonding excursions.

Navigate back to Table 1..

### Table 2. Long description

The table is organized into four columns: Data collection method (duration) and types of questions, Colombia, Nepal, and South Africa.

1. Adolescents section:

- Post session feedback survey (5 min): Not done in Colombia. Nepal had four time points (t 1 n = 12, t 2 n = 11, t 3 n = 8, t 4 n = 8). South Africa had three time points in 2024 (t 1 n = 10, t 2 n = 10, t 3 n = 12).

- Long survey (0.5 h): Colombia had two time points in Dec 2023 (n = 13) and April 2025 (n = 8). Nepal had two time points in May 2024 (n = 11) and June 2025 (n = 12). South Africa had two time points in Feb/May 2024 (n = 9) and June 2025 (n = 6).

- Focus group discussions (F G Ds) or interviews (0.5 to 1.5 h): Colombia had three F G D sessions in Oct/Nov 2024 (n = 6, 6, and 7). Nepal had three F G D sessions in Aug/Sept 2024 (n = 4, 8, and 9). South Africa had one F G D in Nov 2024 (n = 9) and 8 individual interviews in March 2025.

2. Caregivers section:

- Interviews/F G Ds (0.5 to 1.5 h): Not done in Colombia. Nepal had one F G D with n = 10 in June 2025. South Africa had one F G D with n = 9 in March 2025.

3. A A G facilitators section:

- Interviews (0.5 to 1.5 h): Colombia had 1 interview with n = 2 in July/August 2025. Nepal had 1 interview with n = 1 in July 2025. South Africa had 3 interviews with n = 3 in July 2025.

4. Process documents section:

- Record sheets (Oct 2022 to May 2025): Colombia n = 26, Nepal n = 25, South Africa n = 7.

Navigate back to Table 2..

### Table 3. Long description

The table compares survey responses from Colombia, Nepal, and South Africa across two time points, T 1 and T 2.

Header Information:

* Colombia: T 1 n = 13, T 2 N = 8.

* Nepal: T 1 N = 11, T 2 N = 12.

* South Africa: T 1 n = 9, T 2 N = 6.

Section 1: Satisfaction with participation and engagement (Selected Data):

* Purpose of sessions clearly explained: Nepal showed the highest agreement (91% at T 1, 92% at T 2), while Colombia rose from 62% to 75%.

* Could express views freely: Nepal remained at 100% for both time points. South Africa dropped significantly from 88% at T 1 to 24% at T 2.

* Views heard and valued: Nepal was 100% at both points; South Africa increased from 71% to 83%.

Section 2: Impact on adolescents (Selected Data):

* Positive difference in knowledge, skills, confidence, and wellbeing: Nepal started at 100% (T 1) and dropped to 83% (T 2). South Africa increased from 33% to 83%.

* Learn skills and knowledge for the future: Nepal remained high (100% to 92%). Colombia stayed consistent at 54% and 50%.

* Feel enjoyment, satisfaction: Nepal was 100% at T 1 and 92% at T 2. South Africa dropped from 100% at T 1 to 24% at T 2.

* Negative indicators (Stigma, stress, frustration): Nepal reported 0% across all negative indicators at both time points. Colombia and South Africa reported low levels of stress and emotional burden, ranging from 11% to 17%.

Navigate back to Table 3..

### Table 4. Long description

The table consists of four columns: Work packages, Area of influence, Adolescent input and suggestions, and Resulting decision/adaptation.

Work Package 2 (Formative research):

- Privacy and confidentiality: Suggested discreet recording; resulted in journals concealed within books.

- Interview methods: Proposed stories over direct questions; resulted in third-person narrative scenarios.

- Emotional expression: Suggested visual ways to express feelings; resulted in use of emojis.

- Family engagement: Recommended early caregiver involvement; resulted in additional caregiver meetings.

Work Package 3 (Intervention design):

- Family involvement: Suggested parallel caregiver support; resulted in caregiver components.

- Session timing: Proposed alignment with school schedules; resulted in integration into the school day.

- Physical access: Suggested organized transport; resulted in provision of transport.

- Recruitment: Emphasized inclusive participation; resulted in mixed-gender criteria.

- Financial support: Proposed secure payment; resulted in bank account transfers.

- Skills development: Requested more practice; resulted in additional practice time.

- Food provision: Shared food preferences; resulted in culturally appropriate refreshments.

- Physical activity: Suggested local activities; resulted in skating in Colombia and martial arts in Nepal.

Work Package 4 (Instrument development):

- Biometric comfort: Suggested alternative measurement sites; resulted in palm-based heart rate monitoring.

- Survey clarity: Proposed simpler wording; resulted in simplified survey language.

- Concept understanding: Suggested adding examples; resulted in additional explanations.

- Participant fatigue: Recommended rest breaks; resulted in scheduled breaks.

Work Package 5 (Trial implementation):

- Stigma reduction: Suggested non-stigmatizing language; resulted in revised consent materials.

- Eligibility clarity: Recommended clearer messaging; resulted in emphasizing that diagnosis is not required.

- Participant rights: Suggested clearer explanations; resulted in improved rights information.

Navigate back to Table 4..

## References

[r1] Baird S, Choonara S, Azzopardi PS, Banati P, Bessant J, Biermann O, Capon A, Claeson M, Collins PY, De Wet-Billings N, Dogra S, Dong Y, Francis KL, Gebrekristos LT, Groves AK, Hay SI, Imbago-Jácome D, Jenkins AP, Kabiru CW, Kennedy EC, Li L, Lu C, Ma J, McGovern T, Mensa-Kwao A, Mojola SA, Nagata JM, Olumide AO, Omigbodun O, O’Sullivan M, Prost A, Requejo JH, Shawar YR, Shiffman J, Silverman A, Song Y, Swartz S, Tamambang R, Urdal H, Ward JL, Patton GC, Sawyer SM, Ezeh A and Viner RM (2025) A call to action: The second Lancet commission on adolescent health and wellbeing. The Lancet 405(10493), 1945–2022. 10.1016/S0140-6736(25)00503-3. 40409329

[r2] Bakhtiar A, Lang M, Shelley B and West M (2023) Research with and by children: A systematic literature review. Review of Education 11(1), e3384. 10.1002/rev3.3384.

[r3] Daiute C (2016) A relational approach to human development research and practice in 21st century violence and displacement. Human Development 59(2-3), 128–151. 10.1159/000448230.

[r4] Dovey-Pearce G, Walker S, Fairgrieve S, Parker M and Rapley T (2019) The burden of proof: The process of involving young people in research. Health Expectations 22(3), 465–474. 10.1111/hex.12870. 30770609 PMC6543165

[r5] Fereday J and Muir-Cochrane E (2006) Demonstrating rigor using thematic analysis: A hybrid approach of inductive and deductive coding and theme development. International Journal of Qualitative Methods 5(1), 80–92. 10.1177/160940690600500107.

[r6] Fetters MD, Curry LA and Creswell JW (2013) Achieving integration in mixed methods designs-principles and practices. Health Services Research 48(6 pt2), 2134–2156. 10.1111/1475-6773.12117. 24279835 PMC4097839

[r7] Flynn R, Walton S and Scott SD (2019) Engaging children and families in pediatric Health Research: A scoping review. Res Involv Engagem 5(1), 32. 10.1186/s40900-019-0168-9. 31700676 PMC6827239

[r8] Forsythe LP, Ellis LE, Edmundson L, Sabharwal R, Rein A, Konopka K and Frank L (2016) Patient and stakeholder engagement in the PCORI pilot projects: Description and lessons learned. Journal of General Internal Medicine 31(1), 13–21. 10.1007/s11606-015-3450-z. 26160480 PMC4700002

[r9] Forsythe L, Heckert A, Margolis MK, Schrandt S and Frank L (2018) Methods and impact of engagement in research, from theory to practice and back again: Early findings from the Patient-Centered Outcomes Research Institute. Quality of Life Research 27(1), 17–31. 10.1007/s11136-017-1581-x. 28500572 PMC5770504

[r10] Freire K, Pope R, Jeffrey K, Andrews K, Nott M and Bowman T (2022) Engaging with children and adolescents: A systematic review of participatory methods and approaches in research informing the development of health resources and interventions. Adolescent Research Review 7(3), 335–354. 10.1007/s40894-022-00181-w.

[r11] HRA (2017) UK Framework for Health and Social Care Research. Available at https://www.hra.nhs.uk/planning-and-improving-research/policies-standards-legislation/uk-policy-framework-health-social-care-research/uk-policy-framework-health-and-social-care-research/.

[r12] Kok M (2018) Guidance Document: Evaluating public involvement in research. Bristol: University of the West of England.

[r13] Krane V, Klevan T and Sommer M (2021) Youth involvement in research: Participation, contribution and dynamic processes. In Wulf-Andersen T, Follesø R and Olsen T (eds), Involving Methods in Youth Research: Reflections on Participation and Power. Cham: Springer International Publishing, pp. 47–71. 10.1007/978-3-030-75941-4_3.

[r14] Larsson I, Staland-Nyman C, Svedberg P, Nygren JM and Carlsson IM (2018) Children and young people’s participation in developing interventions in health and well-being: A scoping review. BMC Health Services Research 18(1), 507. 10.1186/s12913-018-3219-2. 29954392 PMC6027768

[r15] Lund C, Jordans MJD, Garman E, Araya R, Avendano M, Bauer A, Bahure V, Dua T, Eleftheriou G, Evans-Lacko S, García Rodríguez JF, Gautam K, Gevonden M, Hessel P, Kohrt BA, Krabbendam L, Luitel NP, Roy S, Seifert Bonifaz M, Singh R, Sinichi M, Sorsdahl K, Thornicroft G, Tol WA, Trujillo D, van der Merwe N, Wahid SS and Yarrow P (2023) Strengthening self-regulation and reducing poverty to prevent adolescent depression and anxiety: Rationale, approach and methods of the ALIVE interdisciplinary research collaboration in Colombia, Nepal and South Africa. Epidemiology and Psychiatric Sciences 32, e69. 10.1017/S2045796023000811. 38088153 PMC10803189

[r16] McCabe E, Amarbayan MM, Rabi S, Mendoza J, Naqvi SF, Thapa Bajgain K, Zwicker JD and Santana M (2023) Youth engagement in mental health research: A systematic review. Health Expectations 26(1), 30–50. 10.1111/hex.13650. 36385452 PMC9854331

[r17] Minds Y (2020) Evaluating participation: A guide for professionals. Young Minds.

[r18] Nagata JM, Imbago-Jácome D, Choonara S, Talebloo J, Memon Z, O’Sullivan M, Sawyer SM and Baird S (2025) Reporting of research with adolescent and youth engagement. Lancet Child Adolesc Health 9(7), 442–445. 10.1016/s2352-4642(25)00092-6. 40409336

[r19] NAM (2023) *Participant Questionnaire for the Public and Patient Engagement Evaluation Tool.* National Academy of Medicine. Available at https://nam.edu/product/participant-questionnaire-for-the-public-and-patient-engagement-evaluation-tool/.

[r20] NIHR (2019) UK Standards for Public Involvement; last accessed 31 July 2026.

[r21] O’Brien BC, Harris IB, Beckman TJ, Reed DA and Cook DA (2014) Standards for reporting qualitative rResearch: A synthesis of recommendations. Academic Medicine 89(9), 1245–1251. 10.1097/acm.0000000000000388. 24979285

[r42] Oubiña López, M., & Gómez Baya D. (2025). Interventions to Promote Civic Engagement Among Youth and Its Outcomes on Mental Health: A Scoping Review. Children, 12. 10.3390/children12060665 PMC12191928 40564623

[r22] Palacios-Barrios EE and Hanson JL (2019) Poverty and self-regulation: Connecting psychosocial processes, neurobiology, and the risk for psychopathology. Comprehensive Psychiatry 90, 52–64. 10.1016/j.comppsych.2018.12.012. 30711814

[r23] Patton GC, Olsson CA, Skirbekk V, Saffery R, Wlodek ME, Azzopardi PS, Stonawski M, Rasmussen B, Spry E, Francis K, Bhutta ZA, Kassebaum NJ, Mokdad AH, Murray CJL, Prentice AM, Reavley N, Sheehan P, Sweeny K, Viner RM and Sawyer SM (2018) Adolescence and the next generation. Nature 554(7693), 458–466. 10.1038/nature25759. 29469095

[r24] Pawson R and Tilley N (1997) Realistic Evaluation. London: SAGE Publications Ltd.

[r25] Perowne R, Rowe S, Lajevardi A, Bingham L, Parry E, Grey G, Thomas PC and Gutman LM (2025) Barriers and facilitators to the involvement of under-represented children and Young people (aged 8-25) in mental Health Research - a systematic review. Clinical Child and Family Psychology Review 28(4), 858–881. 10.1007/s10567-025-00544-4. 40983788 PMC12660437

[r26] Preston J, Stones SR, Davies H, Preston J and Phillips B (2019) How to involve children and young people in what is, after all, their research. Archives of Disease in Childhood 104(5), 494–500. 10.1136/archdischild-2018-315118. 31000534

[r27] Rouncefield-Swales A, Harris J, Carter B, Bray L, Bewley T and Martin R (2021) Children and young people’s contributions to public involvement and engagement activities in health-related research: A scoping review. PLoS One 16(6), e0252774. 10.1371/journal.pone.0252774. 34106978 PMC8189547

[r28] Samdal O, Budin-Ljøsne I, Haug E, Helland T, Kjostarova-Unkovska L, Bouillon C, Bröer C, Corell M, Cosma A, Currie D, Eriksson C, Felder-Puig R, Gaspar T, Hagquist C, Harbron J, Jåstad A, Kelly C, Knai C, Kleszczewska D, Kysnes BB, Lien N, Luszczynska A, Moerman G, Moreno-Maldonado C, NicGabhainn S, Pudule I, Rakic JG, Rito A, Rønnestad AM, Ulstein M, Rutter H and Klepp K-I (2023) Encouraging greater empowerment for adolescents in consent procedures in social science research and policy projects. Obesity Reviews 24(S2), e13636. 10.1111/obr.13636. 37753605

[r29] Sellars E, Pavarini G, Michelson D, Creswell C and Fazel M (2021) Young people’s advisory groups in health research: Scoping review and mapping of practices. Archives of Disease in Childhood 106(7), 698–704. 10.1136/archdischild-2020-320452. 33208398

[r30] Staniszewska S, Brett J, Simera I, Seers K, Mockford C, Goodlad S, Altman DG, Moher D, Barber R, Denegri S, Entwistle A, Littlejohns P, Morris C, Suleman R, Thomas V and Tysall C (2017) GRIPP2 reporting checklists: Tools to improve reporting of patient and public involvement in research. BMJ 358, j3453. 10.1136/bmj.j3453. 28768629 PMC5539518

[r31] Stockings EA, Degenhardt L, Dobbins T, Lee YY, Erskine HE, Whiteford HA and Patton G (2016) Preventing depression and anxiety in young people: A review of the joint efficacy of universal, selective and indicated prevention. Psychological Medicine 46(1), 11–26. 10.1017/s0033291715001725. 26315536

[r32] Strauman TJ and Eddington KM (2017) Treatment of depression from a self-regulation perspective: Basic concepts and applied strategies in self-system therapy. Cognit Ther Res 41(1), 1–15. 10.1007/s10608-016-9801-1. PMC5308600 28216800

[r33] Tong A, Sainsbury P and Craig J (2007) Consolidated criteria for reporting qualitative research (COREQ): A 32-item checklist for interviews and focus groups. International Journal for Quality in Health Care 19(6), 349–357. 10.1093/intqhc/mzm042. 17872937

[r34] United Nations (1989) *Convention on the Rights of the Child.* United Nations General Assembly. 20 November 1989, United Nations, Treaty Series. Available at https://www.ohchr.org/en/instruments-mechanisms/instruments/convention-rights-child.

[r35] Warraitch A, Wacker C, Biju S, Lee M, Bruce D, Curran P, Khraisha Q and Hadfield K (2024b) Positive impacts of adolescent involvement in Health Research: An Umbrella Review. An Umbrella Review. J Adolesc Health 75(2), 218–230. 10.1016/j.jadohealth.2024.02.029. 38597838

[r36] Warraitch A, Wacker C, Bruce D, Bourke A and Hadfield K (2024a) A rapid review of guidelines on the involvement of adolescents in health research. Health Expectations 27(3), e14058. 10.1111/hex.14058. 38855830 PMC11163265

[r37] WHO (2014) Health for the World’s Adolescents: A Second Chance in the Second Decade: Summary. Geneva: World Health Organization.

[r38] Wilson O, Daxenberger L, Dieudonne L, Eustace J, Hanard A, Krishnamurthi A, Quigley P and Vergou A (2020) A rapid evidence review of young people’s involvement in health research. London: Wellcome.

[r39] Wong G, Greenhalgh T, Westhorp G, Buckingham J and Pawson R (2013) RAMESES publication standards: Realist syntheses. BMC Medicine 11(1), 21. 10.1186/1741-7015-11-21. 23360677 PMC3558331

[r40] Wyatt KA, Bell J, Cooper J, Constable L, Siero W, Pozo Jeria C, Darling S, Smith R and Hughes EK (2024) Involvement of children and young people in the conduct of health research: A rapid umbrella review. Health Expectations 27(3), e14081. 10.1111/hex.14081. 38845155 PMC11156690

[r41] Young A, Toraif N, Le C, Gergen Barnett K and Augsberger A (2024) Facing power: Navigating power dynamics in a youth participatory action research project situated within a healthcare setting. Journal of Community Practice 32(3), 315–336. 10.1080/10705422.2024.2385624. 39345874 PMC11426412

