# Supplementary material for: Adolescent advisory groups in the ALIVE study: Feasibility and acceptability in global mental health prevention research
Source: Glob Ment Health (Camb). 2026 Jul 20;13:e167. doi: 10.1017/gmh.2026.10274 (PMC13458858; doi:10.1017/gmh.2026.10274)
Supplement: Supplementary file 1 [file S205442512610274Xsup001.docx]

**Supplement: Creative methods description and images**

Appropriate permission was obtained from participants for the use and publication of participant photographs.

| **Activity** | **Images** |
| --- | --- |
| Nepal: Tree diagram, September 2023 | |
| The adolescents were divided into two groups of six. Each group was instructed to brainstorm and develop a cause-and-effect diagram, with “stress among adolescents” as the focal problem. Groups were given 30 minutes to complete the activity. They then presented their diagrams on poster-sized chart paper.  The diagrams identified a range of causes of stress, including family issues, poverty, rejection, unfulfilled aspirations, academic pressure, bullying, low self-confidence, peer conflict, drug use, and discrimination. Reported effects included health problems, anxiety, depression, social isolation, and suicidal thoughts.  The activity aimed to help participants visually map the relationship between problems and their underlying causes, supporting analytical thinking about challenges encountered in daily life. Through this exercise, adolescents practised identifying causes of problems as a step toward developing potential solutions. AAG members indicated that they intended to apply this approach to analyse and address challenges in their own experiences. |  |
| Nepal: Tester of the ALIVE intervention, October 2023 | |
| The session began with a warm-up, using a “hot ball” game to engage participants. This was followed by a check-in on self-reported feelings, a deep-breathing exercise, and a group energizer (“Hip-Hip Hurray”). A brief theoretical introduction on goals and commitments, including examples, was then provided. Participants were divided into two groups, each assigned a separate goal, and completed activities to brainstorm plans and actions to achieve their goals.  This was followed by a martial arts-based physical activity session. The session included a 10-minute warm-up, three 15-minute punching exercises targeting different levels (upper, middle, lower), and a 5-minute cool-down involving bowing and a brief moment of reflection. This was followed by a reflection session for participants to consider how the physical activity helped them progress toward their set goals. An overall reflection on the self-regulation component followed, during which participants established specific goals and action plans for the upcoming month. The session concluded with a check-out activity to capture participants’ experiences.  After a lunch break, a session on “Why Education?” was conducted. Activities included a group exercise exploring the costs and benefits of education across three themes (money, friends, future), a scenario discussion based on a case study, and a video screening with guided discussion. The session concluded with a summary and a brief energizing activity. |  |
| Nepal, Tester of the ALIVE measurements (Body scan and biofeedback), October 2023 | |
| Facilitated warm-up with a jumping jack exercise, followed by a breathing exercise and a check-in using facial expressions. A group energizer (“Hip Hip Hurrah”) preceded a discussion on the effects of stress on the body and mind, including heart rate and breathing. Staff demonstrated the use of a biofeedback device, and one participant volunteered to use it while the group observed. Participants then practiced the Take 5 body scan, reflecting on its calming effects.  This was followed by a martial arts session, beginning with a 6-minute warm-up. Participants reviewed previously learned punching skills and practiced a new “raising block” technique with partners, incorporating breathing exercises. The session concluded with bowing and calming exercises.  Next, the group explored negotiation skills. Participants discussed personal experiences with problem-solving among peers, followed by a Problem-and-Solution card game. Pairs identified corresponding solutions and role-modelled conversations, with group discussion on additional solutions. A structured example statement was used to explore negotiation processes and participants’ reasoning. The arm game was used before and after the negotiation exercise to illustrate competitive versus cooperative strategies.  Finally, participants reviewed a resource map. They confirmed its usability but suggested reducing content to improve readability and accessibility for other adolescents. |  |
| Nepal: World Café and Mental Health Video Session, January 2024 | |
| The adolescents were divided into three groups of four participants each. Participants discussed scenarios in each group for 15 minutes, identifying potential solutions. After each round, participants rotated tables while the group leader remained, ensuring all participants contributed to every scenario. Each table leader then presented a summary of the group discussions.  Scenario 1: Excessive use of TV and mobile phones and its impact on adolescents’ social integration, emotional health, and peer interactions. Participants identified potential negative effects on social engagement and physical and emotional well-being.  Scenario 2: Family conflict and abuse, illustrated through a case study of an adolescent experiencing domestic violence and truancy. Participants suggested preventive strategies, including alerting parents or authorities, and emphasized that suicide is not a solution.  Scenario 3: Home abuse and forced sexual activity. Participants proposed community awareness programs and identified local organizations providing support, including TPO Nepal, Maiti Nepal, and Save the Children.  After the discussions, staff provided additional examples, practical advice, and contact information for support services. Participants expressed intentions to apply this knowledge in daily life and share it with peers and family.  A short documentary on self-awareness and mental health followed. Participants received written summaries and reflected on recognizing and expressing emotions, highlighting the importance of discussing feelings with family and friends. |  |
| Nepal: Art Session and Documentary Video, February 2024 | |
| The session was introduced by outlining objectives, schedule, and AAG members’ responsibilities for participation. Participants received a brief overview of art history, examples of global artists, and guidelines for engagement (A-Act Appropriately, T-Try, R-Respect).  During the art activity, adolescents were provided with plain chart paper and coloured pencils and instructed on basic rules for creating art. They were given 30 minutes to complete their work. Completed pieces were collected and subsequently discussed in the classroom. Adolescents presented their artwork, which addressed topics such as waste management, environmental health, and personal thoughts and feelings. Selected works were discussed in more detail, focusing on participants’ intentions and creative choices.  Participants discussed the benefits of art, including its role in developing creativity, imagination, communication skills, self-esteem, and potential income-generating opportunities. Participants committed to applying the insights from the session in their daily lives and sharing their learning with peers and family.  The session concluded with a short documentary on a blind painter, emphasizing skill development, perseverance, and accepting reality. Participants reflected on the importance of persistence, problem-solving, and supporting others. Participants expressed intentions to incorporate the lessons into daily life and share them with their communities. |  |
| Nepal: Tester of the ALIVE Intervention, *My Supporters + I Start to Save, March 2024* | |
| After energiser, check-in, and overview of objectives, adolescents were introduced to the concepts of social support, identifying supporters, and saving to achieve personal goals. A group activity (“learner and supporter”) was used to illustrate supportive relationships, followed by a debrief on the qualities of effective supporters. Key messages were summarised at the end of the first component.  The planned martial arts-based activity was omitted, and the session proceeded with the “Take 5” exercise. MC then facilitated the second component, focusing on saving behaviours. Small group activities addressed goal setting, reasons for saving, saving methods, and their advantages and disadvantages. Learning maps and saving method cards were used to support discussion and application. The session concluded with a review of key lessons and a group closing activity.  The next session began with an introduction to negotiation principles, followed by a small group activity on problem-solving using a household task-sharing scenario. Full-group activities explored examples of negotiation and the four-step process (“me, you, together, build”). After a short break, adolescents participated in activities on identifying interests and negotiating in challenging situations, including the “babysitter blues” scenario. The session concluded with a discussion of key learning points. |  |
| Nepal: Session on Knowing ourselves, building us, June 2024 |  |
| The session began with an overview of the objectives, schedule, and expectations for AAG member participation. This was followed by a series of structured activities designed to promote engagement, emotional regulation, and group cohesion.  Balloon Game: Participants worked in pairs to keep an inflated balloon from touching the ground. Additional balloons were gradually introduced, and the activity progressed to a full-group format in which all participants shared responsibility. This exercise aimed to encourage cooperation and collective problem-solving.  Check-in and “Take 5”: Participants sat in a circle and were invited to draw freely on post it notes and share the meaning of their drawings. This was followed by a brief guided breathing exercise to support emotional regulation and transition into the main activities.  Getting to Know Each Other: Participants were divided into two groups and engaged in a rapid name-recognition game using a visual barrier. The activity promoted attentiveness and familiarity among group members.  Common Truth and Special Quality: Participants worked in small groups to identify five shared characteristics and one unique quality for each member. These were later shared in the full group, with peers guessing the individual associated with each unique quality. The activity aimed to foster mutual understanding and positive group identity.  Dumb Charades: Participants acted out pre-prepared words in pairs, while others guessed their meaning. This activity encouraged collaboration, communication, and peer support.  Positive Writing: Each participant wore a blank sheet of paper on their back, on which peers wrote positive attributes or qualities they admired. Participants then read and reflected on the feedback, sharing their responses with the group. This activity aimed to promote self-esteem and strengthen peer relationships. |  |
| Nepal: Session on Inner Journey, July 2024 | |
| Facilitators led a discussion on the concepts of spirituality and spiritual health, and their distinction from religion. Most participants initially viewed religion and spirituality as synonymous. The session explored individual spiritual practices (e.g., meditation, prayer, mindfulness, time in nature) and their perceived importance for coping with stress, emotional regulation, and wellbeing.  Participants were guided through a “safe place” imagery exercise to promote relaxation and emotional regulation. They then created drawings representing their understanding of spirituality, which were shared and displayed as a gallery.  Participants engaged in a group movement activity, with each member leading a simple physical action. The exercise aimed to promote body awareness and refreshment.  Facilitators shared a story illustrating personal transformation, responsibility, and generosity. The story was used to prompt reflection on inner growth and purposeful action.  Participants wrote reflective letters to their younger selves, focusing on self-compassion, reassurance, and personal guidance. Sharing was voluntary, and participants reflected on the emotional experience.  Adolescents reported high satisfaction with the session content and delivery. They highlighted the usefulness of the safe place exercise for managing stress and expressed increased awareness of spirituality, self-trust, and self-care. Participants emphasised the importance of dedicating regular time for personal reflection to support overall wellbeing. |      |
| Use of Creative and Reflective Methods in Group Sessions, August 2024 | |
| Session began with movement-based and attention-focused warm-up activities, including structured games and imitation exercises, designed to promote energy, concentration, and group cohesion. These activities encouraged active participation and helped establish a relaxed and inclusive atmosphere.  This was followed by brief check-in and mindfulness exercises, including guided breathing and reflective sharing, to support emotional regulation and create a safe space for engagement.  Creative self-reflection activities were then used to encourage personal expression and peer learning. For example, adolescents completed visual and written templates to represent their interests, strengths, and aspirations, which were subsequently shared within the group. Similarly, drawing-based exercises were used to support goal setting, problem-solving, and forward planning, enabling participants to visually map aspirations, anticipated challenges, and potential solutions.  Sessions concluded with structured grounding and reflection activities. These included sensory awareness exercises and symbolic feedback methods (e.g., selecting colours to represent mood), which supported emotional closure and encouraged participants to reflect on their learning and wellbeing.  Overall, the combination of movement, creative expression, guided reflection, and peer sharing promoted engagement, self-awareness, and mutual support within a supportive group environment. |    |
| Nepal: Use of Participatory Methods in Cyberbullying Sessions, October 2024 | |
| Sessions addressing online safety and cyberbullying were delivered using interactive, group-based learning methods. Facilitators combined guided discussion with collaborative and scenario-based activities to encourage reflection, peer learning, and shared problem-solving.  Group work formed a central component of these sessions. Adolescents worked in small groups using visual materials (e.g., chart paper and markers) to jointly analyse online interactions and develop practical strategies for staying safe. Groups then presented their reflections to peers, supporting communication skills and collective learning.  Narrative and case-based methods were also used to facilitate discussion of sensitive topics. Short, fictional scenarios reflecting common adolescent experiences were presented, followed by guided group reflection. This approach enabled participants to explore emotional responses, social dynamics, and potential support strategies in a low-risk and non-stigmatising way.  Throughout these activities, facilitators encouraged dialogue, mutual respect, and multiple perspectives. The combination of discussion, collaborative tasks, and storytelling supported active participation and helped adolescents engage critically with real-life challenges in a supportive group setting. |  |
| Nepal: Creative and Experiential Activities on Communication and Conflict Resolution, November 2024 | |
| Sessions began with a structured welcome and overview of objectives and participant roles. Engagement was initiated through an improvisational ice-breaker (“What Are You Doing?”), which encouraged spontaneity, attentiveness, and peer interaction through mimed actions and role exchange.  Group-based reflective exercises were then used to support experiential learning. Adolescents completed a structured “conflict reflection” activity, in which they documented personal experiences using guided prompts on emotions, thoughts, behaviours, and alternative responses. Participants shared reflections in small groups, promoting perspective-taking and peer learning.  A collaborative “drawing challenge” was used to explore communication processes. Working in paired groups, participants attempted to reproduce a diagram based on verbal instructions, first with one-way communication and then with two-way interaction. Comparison of outcomes facilitated discussion on listening, clarity, and misunderstanding. Incentives were used to reinforce engagement.  Case-based learning was integrated through short fictional scenarios depicting everyday interpersonal conflicts. These were discussed in facilitated groups, enabling adolescents to practise problem-solving, negotiation, and empathetic communication in a safe setting.  Across activities, facilitators emphasised active participation, reflection, and peer exchange, using games, creative expression, and scenario-based learning to strengthen communication and conflict-resolution skills. |    |
| Nepal: Participatory Research Skills Activities | |
| Sessions began with a structured welcome and brief review of previous learning, during which adolescents shared reflections on earlier activities and presented independently developed research questions and short written assignments. Facilitators supported participants in refining these questions and strengthening their analytical thinking.  An experiential group activity (“research quest”) was then used to introduce core research concepts through practice. Adolescents were organised into small groups and assigned a simple theme, positioning each group as a “research team.” Groups were guided to formulate a research question, collect data from peers using rapid survey techniques, summarise findings visually, and develop brief conclusions using chart paper.  Groups presented their mini-projects to peers, followed by facilitated reflection on methodological challenges, such as time constraints, data collection difficulties, and interpretation of responses. Facilitators supported problem-solving and reinforced key research principles.  Sessions concluded with collective debriefing and feedback, focusing on teamwork, learning processes, and the relevance of research skills to everyday life. Adolescents reported high engagement and increased confidence in applying basic research methods. |      |
| Nepal: Participatory Research Skills Workshop, February 2025 | |
| Sessions began with a brief review of previous learning on research processes and applications, followed by a collaborative “Research Adventure Quest” activity. Adolescents were divided into small, mixed-ability groups, with group composition used intentionally to strengthen participation among less vocal members.  Each group was assigned a published research paper and worked as a “research squad” to identify key components, including the research problem, questions, methods, findings, and conclusions. Groups synthesised their analysis using chart-paper presentations, promoting collaborative learning and visual communication.  Following a short break, groups presented their work and engaged in peer questioning, supported by facilitator-led reflection on teamwork, learning processes, and the relevance of research in everyday life. Small incentives were used to reinforce engagement.  Across activities, emphasis was placed on experiential learning, peer-led analysis, and reflective discussion to strengthen adolescents’ understanding of research and confidence in applying research skills. |    |
| Colombia: Introductory Orientation Session August 2022 | |
| The session began with a movement-based trust-building activity adapted from the TeamUp! methodology (War Child), designed to promote familiarity and group cohesion. Participants were then divided into age groups and engaged in a series of interactive learning activities introducing key ALIVE concepts.  Mental health was explored through body-mapping exercises and guided discussion supported by short film clips. Self-regulation was introduced using movement-based activities and reflective media examples. Poverty and poverty-reduction interventions were discussed through contextualised case studies and role-based scenarios.  These activities were followed by a brief presentation outlining the ALIVE project and the role of the AAGs, supported by visual materials and a discussion of participation incentives. Adolescents were then invited to complete a creative entry task, submitting a short video expressing their interest in joining the AAG.  The session concluded with an open discussion, allowing participants to ask questions and reflect on the activities |  |
| Colombia: Participatory and Creative Engagement Session December 2022 | |

| The session began with a reflective icebreaker using three thematic stations (“panic,” “stress,” and “comfort zone”), where adolescents responded to scenario-based prompts and discussed their emotional reactions. This was followed by a role-play exercise in which participants alternated between “researcher” and “entrepreneur” roles to explore the purpose and value of research through experiential learning.  Adolescents then contributed to the development of the engagement plan through facilitated group discussion, identifying preferred areas and levels of involvement in project activities. Creative self-expression was encouraged through short video recordings, in which participants introduced themselves and reflected on emotional regulation.  A hands-on demonstration introduced physiological monitoring using heart rate variability equipment, supported by visual materials. Participants also reviewed and discussed draft questionnaires, providing feedback on content, acceptability, and feasibility as part of the WP6 protocol development.  The session concluded with a reflective mental health activity using hand-drawing templates, enabling adolescents to express personal goals, interests, and wellbeing priorities through visual and narrative methods. |  |
| --- | --- |
| Colombia: Participatory and Creative Engagement Session March 2023 | |
| The session began with introductions involving AAG members, international researchers, and the Colombian facilitation team. New participants were oriented to the project through peer-led explanations by experienced AAG members, supported by facilitators. An inclusive icebreaker activity was then used to build rapport among all attendees.  A central component of the session was a theatrical role-play exercise, in which adolescents and facilitators were divided into four groups representing different perspectives on youth–parent communication. Through improvised performance and facilitated negotiation, participants explored pathways to wellbeing and worked collaboratively to reach shared agreements. This activity created a structured space for dialogue, reflection, and active participation.  Creative self-expression was further encouraged through a personal mental health testimonial delivered in the form of rap, illustrating the use of artistic media to communicate lived experience and promote wellbeing.  The session also incorporated audiovisual materials, including short video messages from AAG members in Colombia, Nepal, and South Africa, fostering a sense of international connection and shared identity. Participation was formally recognised through the distribution of certificates, reinforcing motivation and sustained engagement. The session concluded with a collective closing and farewell. |  |
| Colombia: Human Body Exercise, September 2023 | |
| The session began with an icebreaker designed to help adolescents feel safe and comfortable engaging in discussion and to introduce the theme of psychosocial support. This activity involved passing a drawing from one participant to another by tracing it on each other’s backs. The exercise supported physical engagement and cognitive activation, helping to energise participants and foster group connection.  Feedback was gathered using a visual emoji-based method, with printed happy, neutral, and sad faces. Each adolescent was invited to select and display the emojis that best reflected their views on how their feedback had been incorporated into the research process. This was complemented by a facilitated round-table discussion, ensuring that all participants had an opportunity to share their perspectives.  A corporal cartography approach was used within the psychosocial support activity. Adolescents were encouraged to represent meaningful aspects of their lives by locating them on different parts of a drawn human body. For example, family relationships might be placed near the heart, while aspirations for travel could be represented in the feet. This visual and embodied method supported reflection and personal expression.  Story-sharing took place in a circular seating arrangement, which promoted inclusivity and mutual listening. Participants explained the significance of the elements they had chosen to represent and reflected on how these related to their lived experiences and identities. |  |
| Colombia: Recreation and Emotion Management Plus Movement Based Exercise, 10 November 2025 | |
| The session began with an icebreaker designed to help adolescents feel comfortable engaging in discussion and interacting with the facilitators.  Participants then completed a reaction and emotional management worksheet, which included sentence prompts such as “When I feel happy, I…,” “When I feel angry, I…,” “My biggest fear is…,” and “The things I do that make me feel better are….” This written activity encouraged individual reflection on emotions and coping responses.  Following this, a movement-based grouping exercise was facilitated. Adolescents were invited to stand and form groups according to different categories announced by the facilitator, such as favourite sport, colour, or animal. The only rule was that no participant could remain alone, requiring individuals to identify similarities and collaborate in forming groups. This activity supported peer interaction and inclusion.  Once groups had been established, participants were invited to share selected responses from their worksheets. This was followed by a guided group reflection focusing on self-regulation, coping mechanisms, and inclusion. The combined use of written, movement-based, and discussion methods supported multiple forms of engagement and expression. |  |
| Colombia: Problem tree analysis July 2024 | |
| The problem tree exercise enabled adolescents to identify the causes, experiences, coping strategies, and barriers related to mental health, while reflecting on differences by gender, age, and cultural background.  Roots (Underlying Causes)  Participants identified multiple sources of mental health stress, including academic and work pressures, financial difficulties, family responsibilities and conflicts, interpersonal relationships, limited opportunities, bullying, insecurity, lack of recreational spaces, substance-use pressure, and wider social and political concerns. Stress was perceived to be greater among older adolescents, those who study and work, and older siblings. Domestic violence was seen as disproportionately affecting girls, while younger adolescents were viewed as more vulnerable to substance-related influences. LGBTQ+ youth and indigenous communities were identified as facing additional structural barriers.  Leaves and Branches (Experienced Impacts)  Adolescents reported experiencing anxiety, depression, sadness, emotional withdrawal, powerlessness, aggression, boredom, and loneliness. Participants perceived that girls experience and express these emotions more openly, while boys are socialised to conceal emotional distress. Younger adolescents were considered to have fewer skills to manage these emotions effectively.  Water (Coping Strategies)  Young people described a wide range of coping mechanisms, including socialising, sleeping, eating, sports, talking to trusted individuals, crying, gaming, writing, meditation, creative activities, and seeking advice. Gender differences were highlighted: girls were seen as more emotionally expressive and likely to seek support, while boys tended to rely more on physical activities and were selective about whom they confided in.  Axe (Barriers to Support-Seeking)  Key barriers included stigma, shame, fear of judgement, lack of family support, limited financial and service access, low mental health literacy, distrust, and weaknesses in the healthcare system. Participants reported that boys face stronger stigma around help-seeking, making it less socially acceptable for them to access professional support.  Trunk (Suggested Areas for Intervention)  Participants recommended that mental health programmes should prioritise safe and trusting spaces, opportunities for open discussion, movement- and sport-based activities, meditation, and psychoeducation. They emphasised the importance of inclusive participation across genders and cultural groups, skilled and supportive facilitators, and varied, engaging activities. Interventions were seen as most effective when they combine promotion, prevention, and early support approaches. |      |
| Colombia: Creative Participatory Engagement, September 2024 | |
| This session combined physical and creative participatory methods to promote engagement, self-expression, and collaboration among adolescents.  The skateboarding activity, drawn from the self-regulation component, provided a dynamic and enjoyable way to encourage physical movement while strengthening peer connections. It supported confidence-building, emotional regulation, and social interaction by creating a shared space for cooperation, mutual encouragement, and informal learning.  The WP6 collage activity used visual and creative methods to enable adolescents to contribute to the development of the lived experiences application. By working with magazines and coloured paper, participants translated their ideas into concrete proposals aligned with the four project pillars. This hands-on approach promoted active involvement, collective problem-solving, and open dialogue, while creating a supportive environment in which young people felt comfortable sharing feedback. Overall, the activity strengthened participant ownership and ensured that adolescents’ perspectives meaningfully informed project development. |  |
| Colombia: Mural-making to Express Mental Health November 2024 | |
| This session used a participatory mural-making approach as a creative method to support adolescent engagement, self-expression, and collective reflection on mental health.  Following an icebreaker to create a relaxed and welcoming atmosphere, participants worked with a mural artist who introduced basic techniques in visual design and painting. Adolescents then took part in a structured brainstorming and drawing activity, using symbols, colours, keywords, and imagined scenarios to represent their experiences, challenges, and sources of support related to mental health. Through individual sketches and group discussion, participants translated abstract ideas into shared visual concepts.  The creative process enabled young people to explore sensitive topics in a non-verbal and accessible way, helping those who were initially hesitant about their artistic skills to gain confidence. Over time, participants became more engaged, expressed enjoyment in drawing, and requested additional time to develop their work. The activity revealed common themes, including the importance of family and peer support, self-care, sports, anxiety, and depression, which were collectively incorporated into the mural concept.  The session also encouraged collaborative decision-making, with adolescents contributing ideas about mural location, safety, and scheduling. These discussions reinforced participant ownership and ensured that practical and contextual concerns were integrated into planning.  Overall, the mural-making method functioned as both a reflective and co-design tool. It promoted creativity, dialogue, and peer learning, strengthened group cohesion, and created a tangible collective output that represented both struggles and resilience. The approach supported meaningful youth participation and informed future engagement activities by embedding adolescents’ perspectives into project design. |  |
| Colombia: Skateboarding, December 2024 | |
| This session used a guided skateboarding workshop as a creative, movement-based method to promote wellbeing, peer connection, and reflective engagement among adolescents.  The session began with participant introductions and informal sharing, creating a supportive group environment. A professional skateboarding instructor was invited to lead a structured class, introducing basic techniques, safety principles, and progressive skill-building activities. Over approximately two hours, adolescents practiced new skills, experimented with movement, and received personalised guidance, allowing them to build confidence through embodied learning and gradual mastery.  Skateboarding functioned as both a physical and expressive medium, offering participants an enjoyable outlet for stress and a non-verbal way to engage with wellbeing themes. The activity encouraged mutual support, communication, and collective problem-solving, as participants learned from both the instructor and one another. Many expressed enthusiasm for continuing the activity and exploring additional skateboarding spaces, highlighting the motivational impact of the method.  The session concluded with a reflective group gathering, supported by informal conversation and shared food, where participants discussed their experiences and evaluated the role of physical activity within the ALIVE programme. The exchange of bracelets symbolised group identity and sustained commitment, reinforcing emotional connection alongside physical participation.  Participants’ feedback informed practical refinements, including scheduling, location planning, and future activity design. Attention was also given to shared use of public space, with safety and coordination emerging as important considerations.  Overall, the skateboarding workshop operated as a participatory, experiential learning method that combined physical movement, creative self-expression, and collective reflection. It strengthened group cohesion, enhanced wellbeing, and provided a concrete mechanism for youth-led feedback and co-design within the programme. |  |
| Colombia: Business Creation Activity, February 2025 | |
| The session used creative, hands-on methods to engage adolescents in problem-solving, critical thinking, and mindfulness.  It began with a presentation and icebreaker to welcome participants, introduce facilitators, and energize the group.  The core business-creation activity was highly interactive and dynamic. Adolescents were split into groups to identify a problem they wanted to address, map its causes on a poster, link it to a real-world context, identify affected stakeholders, and brainstorm solutions. This method encouraged creative thinking, practical problem-solving, and collaborative decision-making. Using posters allowed participants to visualize ideas, explore multiple perspectives, and engage in discussion in a structured yet imaginative way.  The session concluded with a mindfulness and reflection exercise, using breathing techniques to promote calm, focus, and gratitude, followed by a socialization space where adolescents shared experiences, suggestions, and feedback.  Overall, the session combined creative brainstorming, visual mapping, collaborative problem-solving, and mindfulness practices to foster engagement, innovation, and reflective thinking among adolescents. |  |
| Colombia: Arts-based Reflection and Role Play, March 2025 | |
| This session combined arts-based reflection and role-play to support adolescents’ self-awareness, emotional regulation, and interpersonal skills.  The session began with a short presentation and an energising icebreaker to establish a safe and engaged learning environment. This was followed by the “Power Hand” activity, a creative, body-based method designed to help participants identify and internalise their personal strengths. Adolescents traced the outline of their hands and assigned a positive attribute to each finger, creating a visual and tactile representation of their resources. By linking each strength to a physical gesture, the activity provided a practical grounding technique that participants could use during moments of stress or anxiety. Group sharing further reinforced reflection, validation, and peer learning.  Building on this self-awareness work, the session introduced negotiation skills through structured role-play. After learning key strategies, participants engaged in simulated negotiation scenarios in which they represented opposing viewpoints and were required to reach agreement. This experiential method enabled adolescents to practice active listening, compromise, and collaborative problem-solving in a supportive setting. Role-play created space for experimentation and learning from mistakes, strengthening confidence in managing conflict and communication challenges.  The session concluded with a reflective picnic and informal discussion, creating opportunities for social bonding and collective feedback. Participants were encouraged to share suggestions and explore future engagement with the AAG, reinforcing their role in shaping programme development.  Overall, the session used embodied drawing, symbolic representation, and experiential role-play as creative methods to translate abstract concepts such as resilience and negotiation into concrete, memorable practices. These participatory approaches supported emotional regulation, peer learning, and practical skill development within a supportive group context. |  |
| Colombia: Skateboarding, April 2025 | |
| The session employed movement-based, experiential methods to strengthen adolescents’ emotional resilience, self-regulation, and peer connection in a supportive environment.  It began with a presentation and icebreaker, welcoming new participants and energising the group. The skateboarding activity formed the core of the session. Led by an experienced instructor, adolescents learned basic techniques and practiced tricks in a hands-on, playful way. Falling and retrying was framed metaphorically to teach resilience, risk-taking, and perseverance. The activity was followed by a guided reflection, where participants discussed their emotions, what they learned from challenges, and how these lessons could apply to everyday life. This approach combined physical engagement with emotional reflection, linking embodied learning to self-regulation skills.  A TeamUp game (“Catch the Tail”) was also incorporated. This structured, play-based methodology provided a safe space for teamwork, friendly competition, and emotion management. Adolescents experienced excitement, disappointment, and other emotions through play, allowing facilitators to guide reflection on coping strategies and emotional regulation in a dynamic, interactive way.  Overall, the session combined creative, embodied, and game-based methods—skateboarding, TeamUp games, and reflective discussion—to simultaneously foster physical activity, resilience, emotional regulation, and group cohesion. |  |

| South Africa: Problem tree method (group poster development) – 08 Oct 2022 | |
| --- | --- |
| A structured problem-tree participatory method was used with adolescents working in small groups to explore depression and anxiety among young people as the focal problem. Participants collaboratively identified the central issue, which was positioned as the “trunk” of the tree, before analysing underlying causes and consequences through guided discussion. Causes were mapped as the “roots” of the tree, while consequences mapped on the “branches”.  The AAG was split into 3-4 members per group and working in small groups, members recorded their ideas visually using posters, printed pictures, and drawing materials, encouraging members to be creative with their use of drawing, cutting and pasting, and writing (captions or text).  Each group was invited to the front to paste their posters on the roots and branches of the tree, taking the broader group on a visual tour of their thoughts and views.  The activity facilitated deeper exploration of how adolescents understand the drivers and consequences of mental health challenges within their communities. |    |
| South Africa: Brainstorming on flip-chart and Q&A dialogue: Caregivers, 08 Oct 2022 | |
| A separate session was held with caregivers on the same day to introduce the ALIVE project and orient caregivers to the planned intervention components. Facilitators used simple visuals to support explanations of the intervention arms and to help caregivers understand key processes and concepts that would be part of the programme.  The session was structured around open discussion and facilitator-led question-and-answer dialogue, with caregivers invited to ask questions, raise concerns, and share their views as explanations were provided. Particular attention was given to discussing the cash transfer component and other intervention elements, with caregivers contributing perspectives on acceptability, perceived benefits, and potential concerns in their households and communities.  The use of clear, simple visuals supported comprehension and helped keep the discussion grounded and practical, enabling caregivers to engage with the content. |  |
| South Africa: Brainstorming on flip-chart and Q&A dialogue: Adolescents, 18 Feb 2023 | |
| This session focused on discussing the cash transfer component of the study and introducing the Heart Rate Variability (HRV) device. Facilitators used visual materials to explain how the cash transfer model worked and invited adolescents to share their views regarding possible benefits, ideas on what adolescents and caregivers would use the cash transfer for, and potential risks we need to be aware of.  Members were guided in brainstorming ideas, responding to facilitator questions. Visual aids helped participants follow explanations and supported ongoing clarification throughout the discussion.  Demonstration: The HRV device was then demonstrated through a live, hands-on explanation. Facilitators showed the device to the group, explaining its size, placement on the body, and how it would be worn during participation. A facilitator modelled how the device is attached and worn, allowing adolescents to observe the process from start to finish. The device was passed around so participants could handle it directly, examine its weight and texture, and ask practical questions about comfort and feasibility of using this in a school setting. The physical interaction of the with the device allowed members to actively engage on the feasibility and acceptability of using the device in a school setting. |  |
| South Africa: Intentional doodle and visual brainstorming – 26 June 2023 (Adolescent sessions) | |
| A themed or intentional doodle method was used across two adolescent advisory groups for this session, to explore participants’ perspectives on intervention design and implementation.  After a brief introduction to the discussion topics, facilitators presented adolescents with specific prompts related to facilitator characteristics, participant engagement and retention, and understandings of caregiving roles. Participants were provided with a range of drawing materials, including chart paper, coloured pens, and markers, and were invited to respond creatively to the prompts through drawing, writing, or a combination of both.  Working in small groups, adolescents were encouraged to freely doodle or visually represent whatever came to mind in response to the theme. Facilitators emphasised that there were no correct answers and that drawings could include symbols, keywords, short phrases, or illustrations of their choice. This open format allowed participants time to think individually as well as engaging in group discussion as ideas emerged through their group talks.  Once drawings were completed, each group used their visual outputs as a starting point for conversation. Participants explained the meaning behind their doodles and reflected on the ideas proposed by other members and groups. Facilitators prompted further discussion by asking follow-up questions, encouraging adolescents to elaborate on shared themes and differences in perspectives across groups. The drawings were displayed and discussed collectively, allowing participants to build on one another’s reflections.  The activity created a low-pressure environment for expressing views, enabling participation from both more vocal and quieter members while supporting discussion of topics that might otherwise have been difficult to articulate directly. |      |
| South Africa: Experiential group problem-solving activity (“Flip-it”) – 04 May 2024 (Adolescent session) | |
| This session involved experiential testing of an intervention activity designed to support problem-solving and collaboration skills. Facilitators began by briefly explaining the purpose of the activity and introducing three key steps used in the intervention: identifying the problem, trying possible solutions, and adjusting strategies based on feedback.  In a group of 5, members were asked to stand together on a large sheet placed on the ground and work as a group to flip the sheet over without anyone stepping off. Adolescents were given 10-15 minutes to discuss strategies, test different approaches, and work together to complete the task while facilitators observed group interaction and participation.  After completing the activity, facilitators led a short reflection discussion in which participants shared their experiences, including what worked well, what was challenging, and whether the activity felt engaging and age-appropriate. The exercise allowed adolescents to experience the intervention activity directly and provide practical feedback on clarity, difficulty level, and overall suitability for peers. |  |
| South Africa: Brainstorming and Q&A dialogue – 04 May 2024 (Adolescent session) | |
| In a small group of 4 members, we used a brainstorming and Q&A dialogue approach to explore how ALIVE information and study findings could best be shared with young people. As several new members were present, facilitators began with a visual presentation outlining the ALIVE project, its progress since inception, and the role of the adolescent advisory groups. The presentation ensured that participants had a shared understanding before discussion began.  Following the presentation, facilitators invited open discussion and encouraged adolescents to suggest ways findings could be communicated in youth-friendly and accessible formats. Participants shared ideas collectively, building on one another’s suggestions while facilitators guided discussion through prompts and clarifying questions. A scribe documented key ideas during the discussion, allowing participants to focus on contributing without needing to record responses themselves. |  |
| South Africa: Multiple methods (i.e. visual presentations, role-play and scenario-based brainstorming, rumour-chain exercise) – 21 June 2025 (Adolescent session) | |
| This session used the following methods to engage adolescents:   (1) Visual presentations and wall posters   A visual slide presentation was used to walk participants through key milestones from ALIVE 1.0 and introduce the proposed design of ALIVE 2.0. Wall posters displaying key concepts were placed around the room and referred to during explanations. Content was delivered in English, isiXhosa, and Afrikaans, with facilitators pausing regularly to check understanding and invite questions.   (2) Role-play: Scenario-based discussion and sticky note brainstorming exercise (Rumour chain)   Facilitators introduced a hypothetical scenario (“Zoleka joins ALIVE 2.0 and receives a cash transfer…”) to explore how rumours and misinformation might emerge during programme implementation. Adolescents then worked in pairs to role-play how information could spread within their communities. Facilitators suggested possible roles (e.g., the participant receiving the transfer, a peer, caregiver, or community member) to prompt thinking, after which adolescents chose their own roles to enact. Pairs completed the role play and wrote their examples on sticky notes. These were pasted on the wall posters during feedback, and guided group discussion. Facilitators encouraged participants to respond to and build on one another’s contributions, generating collective reflection on how rumours develop and how misunderstandings or conflict could emerge. This visually created a rumour-chain on the wall.  Lastly, in their smaller groups, members were asked to use sticky notes to brainstorm ideas on risks and ideas to mitigate these risks on sticky notes and posted on the wall posters, generating discussion. |        |
| South Africa: Adapted world café discussion and small group poster development – 27 Sept 2026 | |
| Participants selected one of two discussion topics presented through a slide presentation and visual wall posters: the proposed unblinding strategy for ALIVE 1.0 or screening and recruitment for ALIVE 2.0. Members chose which topic to join, forming small discussion groups. Facilitators introduced guiding questions before groups engaged in peer-led discussion, sharing experiences and perspectives while facilitators supported clarification and translation.  Within groups, adolescents generated ideas through dialogue and recorded their inputs visually using sticky notes and flip-chart posters displayed on the wall. After approximately 10–15 minutes of discussion and visual development, ideas were harvested as each group presented their insights to the wider group. This allowed broader group reflection and comparison across topics, with additional ideas emerging during feedback added to sticky notes and pasted onto the topic boards.  Brainstorming and Q&A dialogue (traffic-light exercise)  As part of the discussion on the proposed unblinding strategy for ALIVE 1.0, facilitators introduced a traffic-light poster displayed on the wall to gather feedback from members. The poster was divided into three colour-coded sections: red (risky or concerning), amber (unclear or requiring further explanation), and green (acceptable or viewed positively).  Adolescents commented on specific elements of the proposed approach, including whether feedback should be delivered through combined group meetings involving adolescents, caregivers, and school staff, or through written communication such as letters. Facilitators prompted discussion by asking participants to consider how each approach might be received within schools and communities and whether particular formats could enhance understanding.  The discussion generated practical suggestions and highlighted concerns related to safety, communication, and managing expectations during the unblinding process. |        |
