# Supplementary material for: Adolescent advisory groups in the ALIVE study: Feasibility and acceptability in global mental health prevention research
Source: Glob Ment Health (Camb). 2026 Jul 20;13:e167. doi: 10.1017/gmh.2026.10274 (PMC13458858; doi:10.1017/gmh.2026.10274)
Supplement: Peer review history

# Reviewer report: Review: Adolescent advisory groups in the ALIVE study: Feasibility and acceptability in global mental health prevention research — R0/PR2

**DOI:** 10.1017/gmh.2026.10274.pr2

**Authors:** Victor Ugo

**Affiliations:** The MHPSS Collaborative, Denmark

1. Thank you for the opportunity to review this manuscript, I think this paper has the potential to make a useful contribution to the discourse on adolescent involvement in mental health research.

2. One thing I liked about the paper is that it doesn’t present adolescent involvement as simple or automatically meaningful. The parts of the paper that covered trust, facilitator effort, stigma, unequal participation, incentives, language barriers, and role confusion felt realistic and honest, and similar to some of the challenges I have seen in other applications of young people’s involvement in mental health research.

3. My main concern is that the paper often goes a bit beyond what the study has really shown, for example, the paper says it provides evidence that adolescent involvement is beneficial, improves the ethical quality of research, and strengthens psychosocial wellbeing, but I don’t think the study, as you have presented it, can quite support all of those claims in that form.

4. What I think the paper shows more clearly is that many adolescents valued taking part, that facilitators were very important in making the groups function, and that the groups seem to have informed parts of the wider ALIVE study, which I think is already useful and worth saying clearly, without needing to push the claims further.

5. I also think the paper needs to be clearer that these groups were not only advisory groups in the typical sense, and they seem to have been involved in reflective activities, skills-building, trust-building, breathing and movement based activities and other psychosocial elements. That seems to have been part of what made the groups valuable, but it also makes interpretation more complicated and it becomes difficult to separate the effect of advisory involvement itself from the effect of being part of a supportive group space over time.

6. So I would encourage you to tighten the central claim of the paper as at the moment it feels like the paper is trying to do three things at once- show feasibility and acceptability, show benefit for adolescents themselves, and show influence on the wider research.

7. I also think the paper needs to more carefully approach the differences across the three sites in Colombia, Nepal and South Africa where the advisory groups were not set up in the same way. For example the group in South Africa appears to have been embedded within an existing youth advisory structure. These are quite important differences that likely shaped who joined, how quickly trust developed, how much participants already understood advisory roles, and what kind of input was possible. I can see that the paper mentions these differences, but I don’t think you fully work through what they mean for interpretation.

8. The part of the paper on adolescents’ contribution to ALIVE is promising, but I would have loved to see more concrete examples that reflected what exactly the adolescents suggested, how it influenced the team’s choices and decisions and why that mattered. So, basically, a more direct articulation of young people’s influence on decisions made during the study.

9. Also the ethics and safeguarding side needs more attention as although the paper mentions consent, assent, privacy, etc, I would have liked to see more on safeguarding protocols and procedures including referral pathways (if you had these), and how the team managed the fact that these groups seem to have functioned as more than just advisory spaces.

10. Lastly, in the discussion, I would encourage more cautiousness with the suggestion that youth participation may need to function simultaneously as a research mechanism and as a psychosocial intervention. I can see why you are moving in that direction, and there is material in the paper that could point that way, but I don’t think the current study can establish that as confidently as the way you have worded it.

# Reviewer report: Review: Adolescent advisory groups in the ALIVE study: Feasibility and acceptability in global mental health prevention research — R0/PR3

**DOI:** 10.1017/gmh.2026.10274.pr3

The manuscript presents an evaluation of youth engagement in the context of the ALIVE study. Overall, the authors have done a strong job reporting data from three distinct locations and addressing the fact that practical adaptions were made in data collection to address country-specific adolescent needs. The manuscript would be strengthened by additional details. Comments follow by section:

1) Author list. Nabeel Petersen is listed as “Interfer”. I am not familiar with this term. Is it meant to be interpreter?

2) Impact statement p.2, line 14 the term Adolescent Advisory Group is spelled with title case and below in the abstract is written in lower case. Suggest consistency with this.

3) Abstract p.2, line 47 should this be t1: n=33 and t2 vs. just t=26?

4) Introduction p.3, line 17 I might suggest some more updated citations (the UN citation is from 1989). A couple of ideas:

Samdal O, Budin-Ljøsne I, Haug E, Helland T, Kjostarova-Unkovska L, Bouillon C, Bröer C, Corell M, Cosma A, Currie D, Eriksson C, Felder-Puig R, Gaspar T, Hagquist C, Harbron J, Jåstad A, Kelly C, Knai C, Kleszczewska D, Kysnes BB, Lien N, Luszczynska A, Moerman G, Moreno-Maldonado C, NicGabhainn S, Pudule I, Rakic JG, Rito A, Rønnestad AM, Ulstein M, Rutter H, Klepp KI. Encouraging greater empowerment for adolescents in consent procedures in social science research and policy projects. Obes Rev. 2023 Sep;24 Suppl 2:e13636. doi: 10.1111/obr.13636. PMID: 37753605.

Warraitch A, Wacker C, Bruce D, Bourke A, Hadfield K. A rapid review of guidelines on the involvement of adolescents in health research. Health Expect. 2024 Jun;27(3):e14058. doi: 10.1111/hex.14058. PMID: 38855830; PMCID: PMC11163265.

5) Introduction p.3, line 31. It is not clear to the reader why the UK is singled out in this sentence. I anticipate it is because funding for ALIVE comes from the UK, but this is not apparent at this point in the introduction. I might update this sentence to read, “In some high income countries, national funders require involvement as a core part of the research process”. ---this is also true in the US (Forsythe L, Heckert A, Margolis MK, Schrandt S, Frank L. Methods and impact of engagement in research, from theory to practice and back again: early findings from the Patient-Centered Outcomes Research Institute. Qual Life Res. 2018 Jan;27(1):17-31. doi: 10.1007/s11136-017-1581-x. Epub 2017 May 12. PMID: 28500572; PMCID: PMC5770504.)

6) Introduction p.3, line 38. I appreciated the Lund reference, but I might call this out more explicitly in a sentence. For example, “Detailed methods for the ALIVE study have been previously published.” Also, can you provide the specific study years and target study sample age and size?

7) Methods p.4, line 40. Do you mean descriptive purposes?

8) Methods p. 5, line 54. Can you provide details on the translation and validation of survey questions? Also, can you clarify how surveys were administered? Paper and pencil? Computer?

9) Methods p. 5, line 56. Can you provide details on who developed the interview guides? Were questions developed with adolescent input? For focus groups—how long did they last? Who ran them? Similarly for interviews -how long, who ran them? I see some of this is in the tables, but it would be helpful to have stated in the methods. I assume focus group and interview data were audio recorded, but this is not specifically stated.

10) Methods p.6, line 21. Data process and analysis should include something about translation and if the final data was all analyzed in English following translation. For qualitative analysis, details on coding the transcripts, the number of coders, and how results from different countries were synthesized would be helpful.

11) Results p.7, line 8. For each of the quotes can you indicate if what is presented was translated, e.g., translated from Spanish?

12) Results p.7, line 46 would remove interpretation “highlighting the ethical tension” and end the sentence with “participants.”

13) Discussion p.12, line 55. The authors use the term “low-resource” in the discussion and low/middle income in the introduction. I would suggest consistency and that the term “low-resource” may be preferred. I anticipate some of the findings here may also apply to youth in low-resource settings in high-income countries.

14) Discussion needs a limitation setting. Consider highlighting the challenges to integrate data from different settings and languages where practical adaptations made to support adolescent partner needs may have limited traditional methodologic rigor.

15) Discussion p. 13, lines 20-25. After reading this I went back to the results to be sure these were addressed. I found the quote from the South Africa group re: language barriers. The mistrust in Colombia and additional facilitation in Nepal did not clearly come through in the results. I would make sure these items are discussed in the results section if you are referring to them in the discussion (alternately, you can switch out examples with ones currently in the results).

16) Table 2 AAG facilitators interview. As you have done for adolescent interviews, please indicate if these were in person and how long they lasted.

17) Table 3 it is unclear why some rows are in gray.

18) Please define your abbreviation in the table header “Work Package (WP)”

19) Supplement: The pictures of adolescents are awesome. I would include a statement in the supplement that you have permission to publish participant pictures.

20) In the supplement, the word “Taster” is used. Is this meant to be tester instead?

# Reviewer report: Review: Adolescent advisory groups in the ALIVE study: Feasibility and acceptability in global mental health prevention research — R0/PR4

**DOI:** 10.1017/gmh.2026.10274.pr4

The authors of this manuscript describe a mixed methods study evaluating the implementation of an adolescent advisory group model across multiple resource-limited countries in support of the ALIVE study. The purpose of the manuscript was to highlight successes and opportunities in AAG implementation. The authors acknowledge the challenges and flexibility required of this type of investigation and describe the most notable themes and examples found across AAG implementation locations. Generally, this article is easy to understand and fills a gap of adolescent engagement in research occurring in resource-limited countries. They raise interesting points related to developing an understanding of research and the purpose of AAG involvement as foundational work needed prior to youth fully engaging. I would have liked to see additional comparisons made between implementing in resource-limited countries vs. higher resourced countries. There is significant literature available for the latter to support this comparison. I have included additional comments regarding tables below:

--Table 1: missing information in Nepal, period over which meetings took place

--Table 3: What does the shading mean on some of the rows later in the table?

--Table 3: This table is very busy. I defer to the stylistic preferences of the journal, but perhaps the following format for cells would make it easier to follow: T1: 8 (62%)

--Table 4: Include a brief explanation of what each WP covered in column 1 to create context for the area of influence labeled in column 2.

# Editor report: Recommendation: Adolescent advisory groups in the ALIVE study: Feasibility and acceptability in global mental health prevention research — R0/PR5

**DOI:** 10.1017/gmh.2026.10274.pr5

**Affiliations:** Sociomedical Sciences, Columbia University Mailman School of Public Health, United States

No accompanying comment.

# Editor report: Decision: Adolescent advisory groups in the ALIVE study: Feasibility and acceptability in global mental health prevention research — R0/PR6

**DOI:** 10.1017/gmh.2026.10274.pr6

**Affiliations:** Johns Hopkins University Bloomberg School of Public Health, United States

No accompanying comment.

# Reviewer report: Review: Adolescent advisory groups in the ALIVE study: Feasibility and acceptability in global mental health prevention research — R1/PR8

**DOI:** 10.1017/gmh.2026.10274.pr8

**Authors:** Victor Ugo

**Affiliations:** The MHPSS Collaborative, Denmark

Thank you for your response to the comments

# Editor report: Recommendation: Adolescent advisory groups in the ALIVE study: Feasibility and acceptability in global mental health prevention research — R1/PR9

**DOI:** 10.1017/gmh.2026.10274.pr9

**Affiliations:** Sociomedical Sciences, Columbia University Mailman School of Public Health, United States

No accompanying comment.

# Editor report: Decision: Adolescent advisory groups in the ALIVE study: Feasibility and acceptability in global mental health prevention research — R1/PR10

**DOI:** 10.1017/gmh.2026.10274.pr10

**Affiliations:** Johns Hopkins University Bloomberg School of Public Health, United States

No accompanying comment.
